# The Cocktail Effects on the Acute Cytotoxicity of Pesticides and Pharmaceuticals Frequently Detected in the Environment

**DOI:** 10.3390/toxics12030189

**Published:** 2024-02-28

**Authors:** Balázs Göbölös, Rózsa E. Sebők, Gyula Szabó, Gergő Tóth, Sándor Szoboszlay, Balázs Kriszt, Edit Kaszab, Judit Háhn

**Affiliations:** 1 Department of Environmental Safety, Institute of Aquaculture and Environmental Safety, Hungarian University of Agriculture and Life Sciences, 1 Páter Károly u., 2100 Gödöllő, Hungary; gobolos.balazs@uni-mate.hu (B.G.); rozsa.sebok@gmail.com (R.E.S.); szoboszlay.sandor@uni-mate.hu (S.S.); kriszt.balazs@uni-mate.hu (B.K.); kaszab.edit@uni-mate.hu (E.K.); hahn.judit@uni-mate.hu (J.H.); 2 Department of Environmental Toxicology, Institute of Aquaculture and Environmental Safety, Hungarian University of Agriculture and Life Sciences, 1 Páter Károly u., 2100 Gödöllő, Hungary; gyula.szabo.mate@gmail.com

**Keywords:** *Aliivibrio fischeri*, combination index, carbamazepine, NSAIDs, terbuthylazine, S-metolachlor, tebuconazole, PERMANOVA

## Abstract

Xenobiotics never appear as single, isolated substances in the environment but instead as multi-component mixtures. However, our understanding of the ecotoxicology of mixtures is far from sufficient. In this study, three active pharmaceutical ingredients (carbamazepine, diclofenac, and ibuprofen) and three pesticides (S-metolachlor, terbuthylazine, and tebuconazole) from the most frequently detected emerging micropollutants were examined for their acute cytotoxicity, both individually and in combination, by bioluminescence inhibition in *Aliivibrio fischeri* (NRRL B-11177). Synergy, additive effects, and antagonism on cytotoxicity were determined using the combination index (CI) method. Additionally, PERMANOVA was performed to reveal the roles of these chemicals in binary, ternary, quaternary, quinary, and senary mixtures influencing the joint effects. Statistical analysis revealed a synergistic effect of diclofenac and carbamazepine, both individually and in combination within the mixtures. Diclofenac also exhibited synergy with S-metolachlor and when mixed with ibuprofen and S-metolachlor. S-metolachlor, whether alone or paired with ibuprofen or diclofenac, increased the toxicity at lower effective concentrations in the mixtures. Non-toxic terbuthylazine showed great toxicity-enhancing ability, especially at low concentrations. Several combinations displayed synergistic effects at environmentally relevant concentrations. The application of PERMANOVA was proven to be unique and successful in determining the roles of compounds in synergistic, additive, and antagonistic effects in mixtures at different effective concentrations.

## 1. Introduction

Each year, a significant proportion of the approximately 2.3 billion tons of synthetic chemicals used globally, and 300 million tons used in the European Union, ultimately enters natural waters, which form the basis of our drinking water [1,2]. Approximately one-fifth of the world’s population lacks access to safe drinking water of sufficient quality. Although the chemicals released into the environment, in the majority of cases, are present at very low concentrations (µg/L or lower), their persistent presence represents long-term exposure to the elements of the ecosystem. Many of them may exert a range of chronic effects, such as genotoxicity/mutagenicity, carcinogenicity, teratogenicity, immunosuppressive effects, and endocrine disruption, in addition to acute toxicity, especially when present as components of complex mixtures [3,4].

Among the emerging micropollutants (EMPs), pesticides and pharmaceutical residues stand out for their widespread occurrence and highly diverse biological effects. The annual global use of pesticides is estimated to be around 3 million tons [5]. Concurrently, projections indicate that spending on medicines and will reach USD 1.6 trillion and 3335 billion doses (DDD—defined daily dose, the dose defined by the WHO for a given active substance) in 2024 [6]. This highlights the substantial impact of these chemicals on the environment and underscores the need for comprehensive understanding and management of their presence and effects. Pesticides are predominantly released into the environment through agricultural and horticultural applications. They can leach into deeper soil layers due to precipitation and ultimately enter groundwater or surface waters through agricultural run-off. There is an extensive body of literature, including thousands of studies, documenting the environmental occurrence of pesticide substances. These substances have been detected in every environmental compartment, from Antarctica to the Arctic, and even in rainwater. In the European Union, terbuthylazine (TRB) has emerged as one of the most frequently detected herbicides, replacing the banned atrazine [7,8,9,10,11,12,13]. Terbuthylazine is commonly used in combination with metolachlor (MTC), which is also frequently identified as a residue [14,15,16]. Tebuconazole (TBZ), a triazole fungicide, has gained increasing importance, mainly applied to cereals and grapes. In 2022, it was ranked as the fifth most widely marketed pesticide in Hungary, following metolachlor in the third position and terbuthylazine in the fourth position [17]. These compounds frequently occur together in environmental matrices, mainly in surface and ground waters. Terbuthylazine is most commonly used in combination with S-metolachlor in the EU to control broad-leaved weeds and annual grasses on both agricultural and non-agricultural soils [18]. Alongside terbuthylazine and metolachlor, tebuconazole is being detected in environmental compartments and wastewaters with increasing frequency [19,20,21,22].

Continuously used and released from wastewater treatment plants, pharmaceuticals and pesticides are considered to be pseudo-persistent contaminants. Once they enter the environment, they can persist in their original form or in structurally similar transformation products [23]. The number and concentration of pesticides and their concentrations peak during and after the excessive agricultural application of said compounds in late spring and summer in water bodies [24]. Čelić et al. (2021) reported the co-occurrence of carbamazepine, metolachlor, tebuconazole, and terbuthylazine in all of the samples taken from the Ebro River [25]. Xu et al. (2019) detected carbamazepine (0.02–4.34 ng/L), ibuprofen (0.70–22.91 ng/L), and tebuconazole (0.58–50.04 ng/L) in surface watersheds [26]. Ibuprofen, diclofenac, metolachlor, and tebuconazole have been detected in Lake Guaíba [27]. S-metolachlor, tebuconazole, and terbuthylazine have been detected in Lake Balaton and its sub-catchment area [28]. The main pathways for active pharmaceutical ingredients (APIs) entering surface waters are through domestic wastewater, mainly due to inadequate removal of micropollutants during wastewater treatment processes. In recent years, increasing attention has been given to the monitoring of biologically active chemical residues in our environment [29,30]. On a global scale and within the EU, 771 and 596 APIs have been detected in environmental matrices between 2010 and 2016, respectively. Among them, two nonsteroidal anti-inflammatory drugs (NSAIDs), diclofenac (DCF) and ibuprofen (IBU), and the antiepileptic carbamazepine (CBZ) were the most frequently detected APIs in surface water, groundwater, and drinking water, with 2441, 2363, and 1686 positive hits, respectively [31].

While there is a rich literature available on the toxicology and ecotoxicology of these chemicals, there is a significant research gap regarding the cocktail effects of pesticides and APIs, despite their extremely frequent co-occurrence in the environment [14,32,33].

The investigation of the ecotoxicological effects of mixtures is an arduous task, even though numerous studies on mixture toxicity have been published in recent years. In 2007, Belden et al. reviewed the results of 303 experiments from 45 publications on the cocktail effects of pesticides [34]. In 2014, Cedergreen reviewed 194 two-component pesticide mixtures, 21 metal ion mixtures, and 136 antifoulant mixtures [35]. Martin reported in his 2023 review that there were 761 different publications on mixture toxicology between 2007 and 2017. At the same time, nearly two-thirds of these experiments investigated the effects of binary mixtures [36].

The vast number of possible mixtures makes it nearly impossible to assess every mixture experimentally. Therefore, modeling can be a key approach to assessing the toxic properties of mixtures. However, current modeling methods also have their limitations in practical application. Nowadays, there are two widely used and prominent reference models, concentration addition (CA) [37] and independent action (IA) [38], for predicting the combined effect of chemical mixtures; however, they are only suitable for the additive effect of mixtures. CA assumes that mixture components have the same or similar mode of action (MOA) [37], whereas IA assumes they have a different or dissimilar MOA [38]. According to Cedergreen et al. [39], the use of IA and CA to predict the cocktail effect of binary mixtures resulted in approximately 20% (of 158 mixtures), and 10% accuracy. The Chou–Talalay method (combination index method) is one of the most widely used methods for detecting and quantifying synergistic interactions between two or more chemicals, having been cited over 7000 times over the past few decades [40]. While there have been an increasing number of studies assessing the mixture toxicity of APIs [41] or pesticides [34], the interactions between them have been studied only by a few researchers, applying, in most cases, binary mixtures [42,43].

Considering that the chemicals can form a practically infinite number of combinations, quantitative structure–activity relationship (QSAR) models have been extensively used in forecasting not only the activity of single chemicals but the combined effects of components in mixtures [44]. Still, most of the QSAR models are feasible only for binary combinations and additive toxicities of mixtures [45,46,47,48,49]. The QSAR models are limited by the accuracy of the dataset used to train them. The data available are typically composed of single or few experimental values, which may capture complex biological systems poorly. Overall, the currently available workflow for the analysis of mixture toxicity with QSAR is insufficient and limited. For the more accurate use of QSAR models, there is a need for more reliable, and in general, more experimental data to incorporate [50,51].

Following the whole-mixture or top-down approach, in vitro assays can be used to determine the overall toxicity triggered by complex mixtures and are also widely employed to identify previously unknown effects [52].

The objective of this study was to assess the acute cytotoxicity of the most frequently detected pesticides and pharmaceuticals, namely, metolachlor, tebuconazole, terbuthylazine, carbamazepine, diclofenac, and ibuprofen. We sought to examine their individual impact as well as the mixture effects of their binary, ternary, quaternary, quinary, and senary mixtures using the acute *Aliivibrio fischeri* assay. The synergistic, additive, and antagonistic effects between the chemicals in different mixtures at various effective concentrations were determined using the combination index (CI) method. Furthermore, we aimed to define the role of each compound in the cocktail effects using statistical analytical methods.

## 2. Materials and Methods

### 2.1. Chemicals and Stock Solutions

Active ingredients of pesticides and pharmaceuticals were purchased from Sigma-Aldrich Ltd. (Budapest, Hungary). For toxicity experiments, 20 mg/mL carbamazepine (Supelco^®^, Budapest, Hungary, CAS 298-46-4, purity ≥ 99%), diclofenac-sodium (Supelco^®^, Budapest, Hungary, CAS 15307-79-6, purity ≥ 98.5%), ibuprofen (Sigma-Aldrich^®^, Budapest, Hungary, CAS 15687-27-1, purity ≥ 98%), S-metolachlor (Pestanal^®^, CAS 87392-12-9, purity 98.4%), tebuconazole (Supelco^®^, Budapest, Hungary, CAS 107534-96-3, neat), and terbuthylazine (Pestanal^®^,Budapest, Hungary, CAS 5915-41-3, purity 99.4%) stock solutions were prepared in dimethyl sulfoxide (DMSO, CAS 67-68-5, purity ≥ 99.99%, Fisher Scientific, Budapest, Hungary). For the mixtures, stock solutions containing the active ingredients were mixed in the same proportion (1:1 ratio) (from binary to senary). Additional information about the used pesticides and pharmaceuticals can be found in Table S1.

### 2.2. Aliivibrio fischeri Acute Bioluminescence Assay (Microtox^®^)

To determine the acute cytotoxicity of pesticides, APIs, and their mixtures, a standard Microtox^®^ acute assay was performed using the bioluminescence *Aliivibrio fischeri* (AVF) (DSM-7151, NRRL B-11177) test organism. A decrease in light emission due to any negative changes in the metabolic status of the cells is easily detectable, and the results obtained are highly reproducible. The test is often used as the first screening method, due to its rapid and cost-effective feasibility.

Microtox^®^ acute AVF tests were performed according to ISO 11348-1 (ISO 11348-1:2007 Water quality (https://www.iso.org/standard/40516.html (1998) (accessed on 28 December 2023)). Determination of the inhibitory effect of water samples on the light emission of *Vibrio fischeri* (Luminescent bacteria test) Part1: Method using freshly prepared bacteria. A total of 20 mg/mL stock solutions of carbamazepine (CBZ), diclofenac (DCF), ibuprofen (IBU), S-metolachlor (MTC), tebuconazole (TBZ), and terbuthylazine (TRB) were used in the acute assay, diluted from 100 mg/L to 6.25 mg/L in a 2 *w*/*w*% NaCl solution containing 1 *v*/*v*% DMSO. From binary to senary mixtures, chemicals were combined in equal concentrations and diluted from 200 mg/L to 12.5 mg/L. The solvent control sample was the dilution solution (2 *w*/*w*% NaCl; 1 *v*/*v*% DMSO). The final concentration of DMSO was 0.5 *v*/*v*% in the assay, which is non-toxic to the test organism as described in Tóth et al. (2019) [53], and also did not result in any aberrance in bioluminescence in the negative control after 30 min of exposure, according to the ISO standard. Tests were performed in two parallels with a control and nine different concentrations of the chemicals at 15 ± 0.2 °C. The relative bioluminescence was detected by the Microtox^®^ Model 500 Analyzer (*SDI, Carlsbad, California*) after 30 min of incubation, and bioluminescence inhibition was determined. For each compound and mixture, the effective concentration values resulting in 10, 20, 50, 80, and 95% inhibition in the bioluminescence (EC_10, 20, 50, 80, 95_) were calculated from the concentration-response curves using the MicrotoxOmni^®^ software (version 1.1, AZUR Environmental Corp., Carlsbad, California, USA).

### 2.3. Combination Index (CI) Method to Determine Joint Toxicities

Synergistic, additive, and antagonistic effects for the combinations were characterized by combination index (CI) values at inhibition rates in the bioluminescence of 10%, 20%, 50%, 80%, and 95% (EC_10_, EC_20_, EC_50_, EC_80_, EC_95_, respectively). The CI values were calculated using the following equation, as described by Chou and Yang et al. [54,55]:

$$n_{(CI)x^{=}}\sum_{j=1}^{n}\frac{\left(D_{x}\right)1-n\{\left[D\right]j/\sum_{1}^{n}\left[D\right]\}}{\left(D_{m}\right)j\left\{\left(f_{a}x\right)j/\left[1-\left(f_{a}x\right)j\right]\right\}1/mj}$$

*^n^*(CI)*_x_*—the combination index (CI) for n chemicals at an inhibition rate of x%.
(D_x_)_1−n_—the sum of the concentrations of n chemicals, causing an inhibition rate of *x%* in the mixture.
{[D]j/$\sum_{1}^{n}\left[D\right]$}—the proportionality of the individual concentration of *n* chemical causing an inhibition rate of *x%* in the mixture.
(D_m_)*_j_*{(*f*_a_*x*)_j_/[1 − (*f*_a_*x*)_j_]}1/*mj*—the concentration of each individual chemical causing an inhibition rate of *x*%, where D_m_ is the median-effect concentration (antilog of the x-intercept of the median-effect plot), *f*_a_*x* is the fractional inhibition at *x*% inhibition, and *m* is the slope of the median-effect plot.

The effects of mixtures were classified according to Chou and Talalay [56] as synergistic if CI < 1, additive (concentration addition) if CI = 1, and antagonistic if CI > 1. Chou described a more detailed classification with ranges of combination index and description, which were the following: < 0.1—very strong synergism, 0.1–0.3—strong synergism, 0.3–0.7—synergism, 0.7–0.85—moderate synergism, 0.85–0.90—slight synergism, 0.90–1.10—nearly additive, 1.10–1.20—slight antagonism, 1.20–1.45—moderate antagonism, 1.45–3.3—antagonism, 3.3–10—strong antagonism, >10—very strong antagonism [54].

CompuSyn 1.0 software (ComboSyn, Inc., Paramus, NJ, USA) was used to determine and calculate concentration–response curve parameters (m—quantitative estimation of sigmoidicity and r—regression coefficient) and CI values [49]. To determine synergism, additive effect, or antagonism, 6 concentration-response data points (EC_10_, EC_20_, EC_50_, EC_80_, EC_90,_ and EC_95_) were used for the combinations, consisting of 2, 3, 4, 5, and 6 compounds.

### 2.4. Statistical Analysis—Methods

Statistical analyses were conducted in R Statistical Software (version 4.0.2., R Core Team, 2020) by the following packages: ‘tidyr’, ‘scales’, ‘dtw’, ‘vegan’, ‘ggplot2′, ‘circlize’, ‘ComplexHeatmap’, ‘gridExtra’ (‘RColorBrewer’, ‘ggsci’, ‘colorRamps’, ‘viridis’).

The type and intensity of interaction between chemical components are frequently expressed by combination indices (CIs) ranging from zero (extremely strong synergy) to positive infinity (extremely strong antagonism), where values close to one denote additivity (see Table S5). Distances between the limit values (of steps from strong synergy) to additive effect are relatively similar, while steps from additivity to antagonism are increasing exponentially.

For statistical and visual purposes, the combination indices (CIs) of the compounds calculated by CompuSyn software were transformed and centered as follows [57]:

if x > 1 x’ = (−1) × log10(x), and

x ≤ 1 x’ = 1 − x,

where x refers to the raw value and x’ is the result of transformation (see Table S5, Figure S1).

Euclidean distances were calculated between the 26 samples using the transformed CI values at effect sizes of 10%, 20%, 50%, 80%, 90%, and 95%. To identify the most prominent compounds and compound combinations, the distance matrix was subjected to permutational multivariate analyses of variance (PERMANOVA), where 999 permutations established significance. Multivariate homogeneity of variances (BETADISPER) was also examined using the spatial median to reduce original distances to principal coordinates [58,59,60,61].

In any case, where the cytotoxic effect was not detectable, meaning one-sided simple enhancement or potentiation [62], the enhancement of the non-toxic chemical in a mixture was expressed as a percent of the required dose (mg/L) change of the other compounds in the mixture not containing the non-toxic chemical to result in the same effect size as the mixture containing it. We used the following equation to calculate the enhancing effect (E%) of a non-toxic chemical:

E% = {1 − [(EC_xA_ × n)/(n + 1))/(EC_xB_/n)]} × 100

where

EC_xA_—the effective concentration divided by the number of compounds in the mixture containing the non-toxic component resulting in x% bioluminescence inhibition.
n—the number of the chemicals in the mixture not containing the non-toxic component.
EC_xB_—the effective concentration divided by the number of compounds in the mixture not containing the non-toxic component resulting in x% bioluminescence inhibition.

In these cases, the dose change data at effect sizes of 10%, 20%, 50%, 80%, 90%, and 95% were used for Euclidean distance calculation, and then PERMANOVA and BETADISPER were conducted to identify which compounds or compound combinations were significantly affected by the presence of a non-toxic chemical.

## 3. Results

### 3.1. Cytotoxicity on Aliivibrio fischeri

The effective concentration values of carbamazepine, diclofenac, ibuprofen, S-metolachlor, tebuconazole, and terbuthylazine, as well as their mixtures resulting in 10, 20, 50, 80, 90, and 95% inhibition in the bioluminescence of *Aliivibrio fischeri*, are summarized in Table 1.

The effective concentrations resulting in bioluminescence inhibition in *Aliivibrio fischeri* varied over an extremely large range. Among the APIs, the NSAID ibuprofen and diclofenac had similar cytotoxic effects at lower concentrations; however, diclofenac showed higher toxicity with an increase in concentrations. Carbamazepine had significantly lower toxic effects at 50% effective concentration and above. Among pesticides, tebuconazole induced the highest inhibitions, while terbuthylazine, as described in our previous work [53], was non-toxic at any applied concentrations (up to its solubility limit). At 50% effective concentrations, the binary DCF + IBU, IBU + TRB, and ternary CBZ + DCF + IBU mixtures were the most toxic cocktails, with 12, 18, and 12 mg/L EC_50_ values, respectively. The effective concentration values eliciting 10% inhibition in bioluminescence altered between 2 and 10 mg/L in most cases, but there were cocktails that could cause 10% inhibition only at higher applied concentrations. At 95% effective concentrations, the most toxic mixtures were the binary DCF + IBU and ternary CBZ + DCF + IBU with 62 and 54 mg/L EC_95_ values, respectively. Compared to the effects of single chemicals, the most outstanding interactions, i.e., excessively increased toxicity at all concentrations, were observed when combining diclofenac and ibuprofen, and ibuprofen with terbuthylazine, up to 50% above the original effect due to the potentiation effect of the latter. Mixing the three APIs also resulted in highly increased toxicity compared to the single compounds.

### 3.2. Combination Index Values and Enhancement by Terbuthylazine

Combination indices for the mixtures containing carbamazepine, diclofenac, ibuprofen, S-metolachlor, and tebuconazole are summarized in Table 2.

Overall, synergy was observed in 73% of the mixtures in at least one effective concentration. Three cocktails showed additive toxicity compared to the individual components at EC_50_ or higher concentrations, and only four mixtures (15%) were antagonistic on bioluminescence inhibition in *Aliivibrio fischeri* at all effective concentrations.

In general, synergism appeared and intensified as the concentrations were increased. Regarding the CI values at EC_50_, the strongest synergy occurred between the binary DCF + IBU (CI = 0.525), ternary CBZ + DCF + IBU (CI = 0.399), DCF + MTC + TBZ (CI = 0.511), and CBZ + DCF + MTC (CI = 0.534), while the lowest CI values were produced by the APIs in a ternary mixture, followed by the binary carbamazepine + S-metolachlor pair.

Among the combinations, the binary DCF + IBU, DCF + MTC, and MTC + TBZ and ternary CBZ + IBU + MTC, DCF + IBU + MTC, and DCF + MTC + TBZ mixtures showed synergistic effects in cytotoxicity at all concentrations from EC_10_ to EC_95_.

In two ternary mixtures containing CBZ + IBU + MTC and IBU + MTC + TBZ, synergistic effects could be observed at the lowest effective concentrations, while antagonism appeared with increased concentrations.

The quaternary CBZ + IBU + MTC + TBZ mixture showed moderate synergism at the lowest effective concentration, an additive effect at EC_20_, and antagonism at higher concentrations.

The cytotoxic effect of terbuthylazine alone was not detectable (see Table 1); therefore, its effect combined with the other compounds or compound combinations is not synergism or antagonism but a one-sided simple enhancement, potentiation, or augmentation. Thus, the CI calculation is not applicable in this case [62]. The enhancement of cytotoxicity in a mixture resulting in the presence of terbuthylazine is expressed as a percentage and summarized in Table 3.

In almost all the mixtures, terbuthylazine had a great enhancing effect on the other chemicals at lower effective concentrations, which decreased in parallel with the concentration increase. Only the binary mixture with carbamazepine had no effect on terbuthylazine, while in the ternary mixture, it had an inhibitory effect on the other two pesticides at all concentrations.

Overall, 29 of 31 mixtures containing non-toxic terbuthylazine were more toxic at low (in some cases environmentally relevant) effective concentrations toward *Aliivibrio fischeri* than the individual components.

### 3.3. Statistical Analysis of Synergism or Antagonism between Carbamazepine, Diclofenac, Ibuprofen, S-metolachlor, and Tebuconazole

#### 3.3.1. Pairwise Description

**Figure 1:**
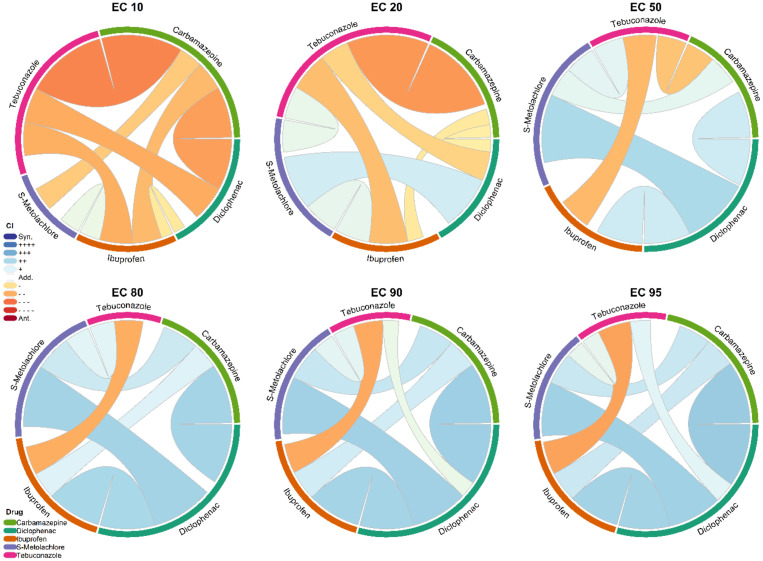
Weighted mean of transformed combination indices of the compound pairs from all the combinations where the chemicals were present in the examined combinations at effect sizes of 10%, 20%, 50%, 80%, 90%, and 95%. The strength of synergy (blue) and antagonism (red) is shown by the width of linkage and color intensity.

The weighted mean of transformed CI values of all combinations where the compound pairs were present, the defining relationships of compound pairs in all the examined combinations, and effect sizes are shown in Figure 1.

Regarding all the mixtures, at 10% effect size, only the pair of ibuprofen and S-metolachlor caused slight synergistic effects; the relationships of the other pairs were additivity or antagonism.

At a 50% effective concentration, tebuconazole had moderate antagonistic relationships with carbamazepine as well as with ibuprofen. At this concentration, the type of relationships of diclofenac in pairs with carbamazepine, with ibuprofen, or with S-metolachlor were moderate synergisms in every mixture, although the latter was close to the bound between synergism and moderate synergism. S-metolachlor also showed slight synergism in combination with carbamazepine or with tebuconazole.

At the 95% effect size, only the pair of ibuprofen and tebuconazole was antagonistic. The types of relationships of diclofenac in pair with carbamazepine, with ibuprofen, or with S-metolachlor were synergies, and with tebuconazole there was a slight synergism. Moderate synergism occurred in the case of carbamazepine combined with ibuprofen or with S-metolachlor. Slight synergism describes the combination of the S-metolachlor and tebuconazole pair.

In Figure 1, the size of the colored bars for each chemical represents the summed absolute values of the strength of synergy and antagonism. At a 10% effect size, carbamazepine and tebuconazole had the largest areas, while at a 50–95% effect size, diclofenac was the most dominant one.

The relationship between the carbamazepine and diclofenac pair was mostly synergistic, especially at effect sizes above 50%, but at 10% effect size, it was antagonism. The carbamazepine and ibuprofen pair was also moderately antagonistic at the 10% effect size, and it changed to moderate synergism with increasing effective concentrations. Most pairs showed a similar tendency along with increasing effect sizes. The most prominent exception was the ibuprofen–tebuconazole pair, which showed antagonism at all effect sizes.

#### 3.3.2. PERMANOVA

According to PERMANOVA, the chosen parameters explain 88.4% of the variance (Table 4). The most significant compounds were tebuconazole (21.95%), ibuprofen (10.69%), and S-metolachlor (8.1%).

**Figure 2:**
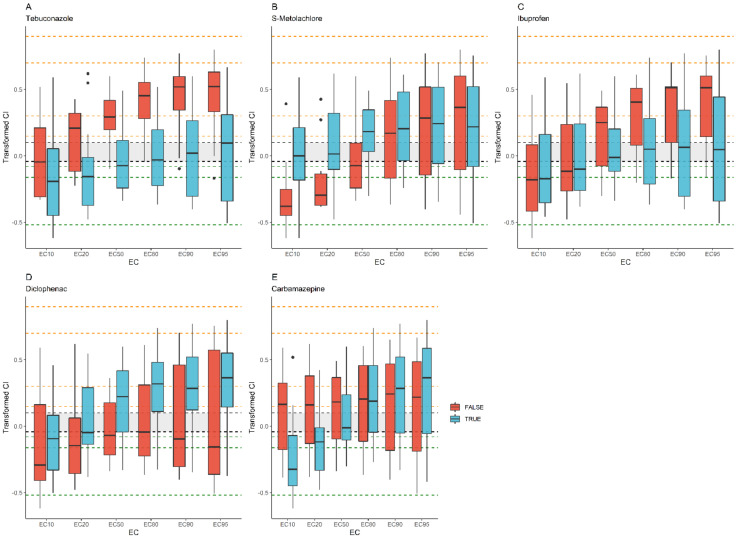
Comparison of transformed combination indices (from 0.5 to −0.5) of samples containing a certain compound (blue) with samples not containing it (red). Dotted lines indicate bounds between synergism (orange lines)–antagonism (green lines) assignments, while the grey band marks additivity. Transformed CI values are indicated in Table S5 in detail. (**A**) Tebuconazole; (**B**) S-metolachlor; (**C**) ibuprofen; (**D**) diclofenac; (**E**) carbamazepine.

Samples containing tebuconazole mainly showed moderate or slight antagonism at 10–20% effect sizes and additivity at 80–95% effect sizes (Figure 2). Ibuprofen affected samples similarly. In both cases, medians were less synergistic across nearly all effect sizes than medians not containing the compound. Samples containing S-metolachlor were mainly additive at 10–20% effect sizes and moderately synergistic at 50–95% effect sizes. Medians of samples not containing S-metolachlor were more antagonistic at 10–20% effect sizes. Samples containing diclofenac showed mainly slight antagonism at 10% effect size and mainly moderate antagonism at 50–95% effect size. In most cases, they were more synergistic at all effect sizes than samples without it. Medians of samples containing carbamazepine showed antagonism, moderate antagonism, or additivity, while medians of other samples were slightly or moderately synergistic at 10–50% effect sizes.

**Figure 3:**
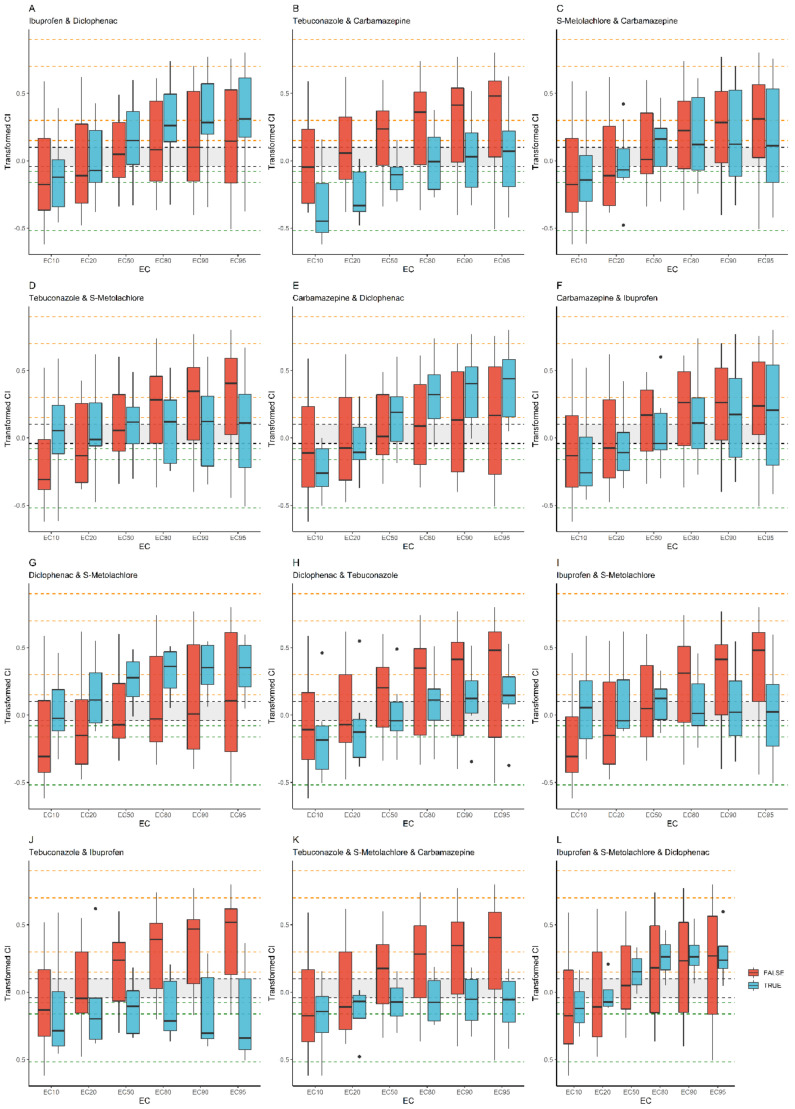
Comparison of transformed combination indices (from 0.5 to −0.5) of samples containing a certain compound combination (blue) with samples not containing it (red). Dotted lines indicate bounds between synergism (orange lines)–antagonism (green lines) assignments, while the grey band marks additivity. Transformed CI values are indicated in Table S5 in detail. (**A**) Ibuprofen and diclofenac; (**B**) tebuconazole and carbamazepine; (**C**) S-metolachlor and carbamazepine; (**D**) tebuconazole and S-metolachlor; (**E**) carbamazepine and diclofenac; (**F**) carbamazepine and ibuprofen; (**G**) diclofenac and S-metolachlor; (**H**) tebuconazole and diclofenac; (**I**) ibuprofen and S-metolachlor; (**J**) tebuconazole and ibuprofen; (**K**) tebuconazole and S-metolachlor and carbamazepine; (**L**) ibuprofen and diclofenac and S-metolachlor.

Interactions of ibuprofen–diclofenac (10.56%), tebuconazole–S-metolachlor (7.58%), and tebuconazole–S-metolachlor–carbamazepine (5.7%) were also remarkable. Medians of samples containing ibuprofen and diclofenac were more synergistic than samples that did not contain this combination (Figure 3). Medians of samples containing the combination of tebuconazole and S-metolachlor were additive or slightly synergistic, while medians of samples not containing this combination were antagonistic at 10% effect size and synergistic at 90–95% effect sizes. Medians of samples containing the combination of tebuconazole, S-metolachlor, and carbamazepine showed slight antagonism, while medians of other samples were at least moderately synergistic at 50–95% effect sizes.

Variances of each parameter included in the model were homogenous, and therefore the significant differences detected by PERMANOVA were the result of a locational effect and not by dispersion (Tables S6 and S7). Distances between samples are represented by nonmetric multidimensional scaling ordinations (stress = 0.0081) by parameters in Figures S2–S4.

### 3.4. Enhancement by Terbuthylazine

#### 3.4.1. Pairwise Description

**Figure 4:**
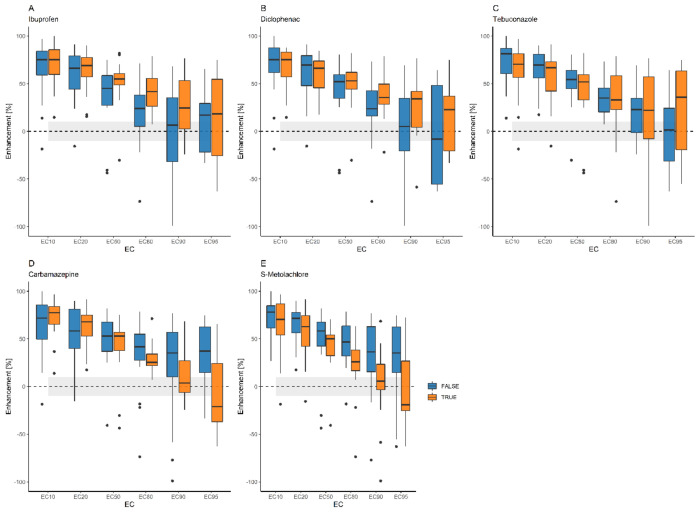
Comparisons of the enhancement (from 100% to −100%) effect by terbuthylazine on samples containing a certain compound (blue) with other samples (red). Grey band marks less than ± 10% enhancement. (**A**) Ibuprofen; (**B**) diclofenac; (**C**) tebuconazole; (**D**) carbamazepine; (**E**) S-metolachlor.

**Figure 5:**
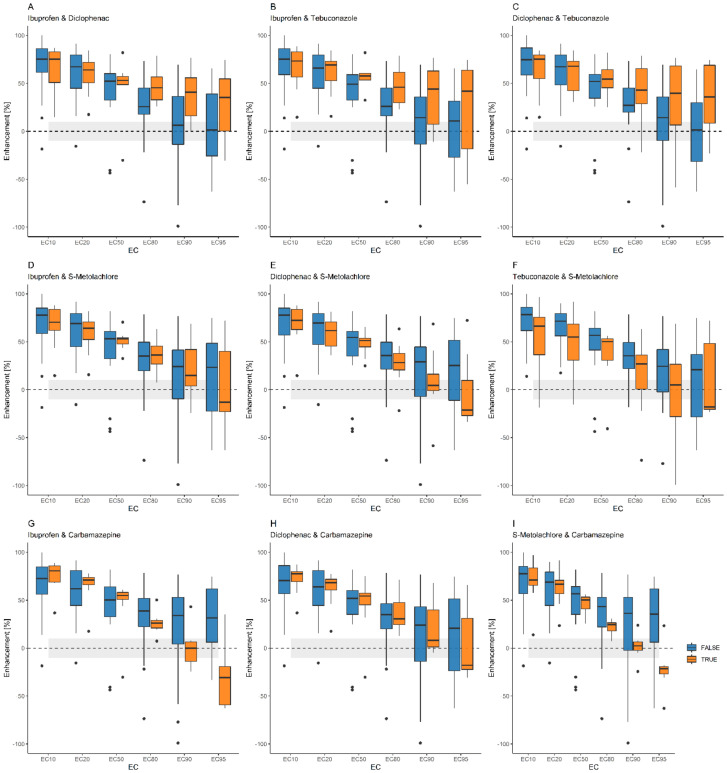
Comparison of enhancement effect (from 100% to −100%) by terbuthylazine on samples containing a certain compound combination (blue) with other samples. Grey band marks less than ± 10% enhancement. (**A**) Ibuprofen and diclofenac; (**B**) ibuprofen and tebuconazole; (**C**) diclofenac and tebuconazole; (**D**) ibuprofen and S-metolachlor; (**E**) diclofenac and S-metolachlor; (**F**) tebuconazole and S-metolachlor; (**G**) ibuprofen and carbamazepine; (**H**) diclofenac and carbamazepine; (**I**) S-metolachlor and carbamazepine.

Effects of most chemicals and chemical combinations were greatly enhanced (over or around 50%) by terbuthylazine at lower effect levels (10–50%). On the other hand, median enhancement was a lesser and lesser characteristic at increasing effect levels. The median enhancement by terbuthylazine was greater with diclofenac above 80% effect levels, with ibuprofen at 80 and 90% effect levels, or with tebuconazole at a 95% effect level compared to other samples. The combination of terbuthylazine with carbamazepine or S-metolachlor resulted in lower median enhancement above 80% effect levels or even reduced the effects at the 95% effect level (see Figure 4 and Figure 5).

#### 3.4.2. PERMANOVA

According to PERMANOVA, samples containing terbuthylazine were most strongly affected by the combination of ibuprofen, diclofenac, and carbamazepine (16.3%); by the combination of ibuprofen and diclofenac (13.7%); by diclofenac alone (11.9%); by the combination of ibuprofen, diclofenac, and tebuconazole (10.97%); by ibuprofen alone (9.8%); by the combination of diclofenac and carbamazepine (7.2%); by carbamazepine alone (6.8%); and by the combination of diclofenac and tebuconazole (5.7%). The effect of tebuconazole alone was also significant, but it explained only 1.7% of the variance. Further significant combinations with smaller proportions of explained variance are also listed in Table 5.

Variances of each parameter included in the model were homogenous, and therefore the significant differences detected by PERMANOVA were the result of the locational effect and not by dispersion (Tables S6–S11). Distances between samples are represented by non-metric multidimensional scaling ordinations (stress = 0.0050) by parameters in Figures S5–S7.

## 4. Discussion

Among the arsenal of micropollutants, active pharmaceutical ingredients and pesticides are one of the greatest ecotoxicological concerns, especially when they are occurring as an unintentional mixture in the environment derived from many different sources at varying doses.

In our work, we experimentally assessed the cocktail effects between three APIs and three pesticides from the most frequently detected micropollutants, creating all the possible combinations in the acute (30 min) *Aliivibrio fischeri* ecotoxicological assay. Interactions between the compounds in non-equitox mixtures were determined by using the combination index method [40]. Moreover, going beyond the sheer characterization of interactions between the compounds by combination indices, we uniquely used a permutational multivariant analysis of variance (PERMANOVA) to determine the roles of the compounds in the mixtures containing pharmaceuticals and pesticide with different modes of action.

While these compounds were developed to affect specific biological processes in humans, animals, and plants, they also have an effect on non-target organisms in the ecosystem. The exact effects how these compounds with varying mode of actions impact the non-target organism is still unclear. Furthermore, there is a possibility of various unpredictable chemical interactions between these compounds at the molecular level, influencing toxicity [63,64]. Ecotoxicological testing is necessary to assess the cumulative biological effects of chemical cocktails including the changed biological effects due to molecular reactions between the compounds.

The *Aliivibrio fischeri* bioluminescence assay serves as a sensitive, easy, and reproducible method for evaluating the general cytotoxic effects of chemicals and environmental samples. Several studies have been conducted on binary mixtures of pharmaceuticals, including diclofenac and ibuprofen. Noteworthy among these are investigations by Di Nica et al., Ukić et al., Zuriaga et al., and Drzymała et al., who reported a relatively high incidence of synergy and additive effects [65,66,67,68]. In contrast, studies by Ge et al. (2020) [69], focusing on the interaction between antibiotics in binary mixtures, did not observe synergic effects; instead, they reported antagonism or additive actions. Additionally, Cedergreen et al. (2006) and Sigurnjak et al. (2020) delved into the effects of binary mixtures of pesticides [70,71] and frequently identified additive action and synergy between pesticide pairs using the *Aliivibrio fischeri* assay. These findings underscore the assay’s utility in discerning interactions and effects within various chemical mixtures. Assessing the interactions between pesticides and antibiotics, Baek et al. (2019) registered synergy only in 12% of the mixtures, while Matias et al. (2023) found high synergistic incidence in those containing prochloraz [42,72]. Villa et al. (2012) investigated the effect of eight complex mixtures with a high number of components (up to 84 chemicals) on *Aliivibrio fischeri* but found that none of the interactions were a synergy [73]. Jacob et al. (2020) examined four pharmaceuticals (metformin, simvastatin, diazepam, and omeprazole) and all their possible mixtures, detecting only antagonism [74]. Białk-Bielińska et al. (2022) conducted experiments on *Aliivibrio fischeri* with a mixture of three NSADs (diclofenac, ibuprofen, and naproxen) and three antibacterial drugs, registering mostly additive actions between the APIs [43].

According to our results, in all the mixtures not containing terbuthylazine, the most significant effects were induced by tebuconazole, ibuprofen, and S-metolachlor. While tebuconazole and ibuprofen generated additivity only at higher effective concentrations, S-metolachlor (with low toxicity on itself) provoked additivity and synergy at all concentrations. Tebuconazole and S-metolachlor together were also acting additively or synergistically. Mixtures containing terbuthylazine were highly enhanced, especially at lower effective concentrations.

Previous reviews highlighted that synergy is a fairly rare phenomenon in mixtures [35,36]; however, in our study, synergistic effects occurred in more than 70% of the cocktails in at least one effective concentration, and non-toxic terbuthylazine had a toxicity-enhancing effect in all mixtures but two. Moreover, while most of the mixtures that we tested showed increased synergy in proportion to the increased concentration, ibuprofen and tebuconazole paired with carbamazepine or S-metolachlor showed synergy at low, environmentally relevant concentrations.

Similarly to previous studies [65,66], our results showed the synergistic effects of diclofenac on other pharmaceuticals; however, DCF also had a toxicity-increasing effect on pesticides. PERMANOVA analysis (based on the CI values), uniquely used for this purpose in our study, confirmed that both diclofenac and carbamazepine, alone and together, acted synergistically in the mixtures. Diclofenac also resulted in significant synergism paired with S-metolachlor and in a ternary mixture with ibuprofen and S-metolachlor. S-metolachlor alone and paired with ibuprofen or diclofenac increased the toxicity at lower effective concentrations in the mixtures.

Due to the ineffective techniques for the removal of micropollutants and the lack of force of legislation regarding these compounds, both APIs and pesticides are often detected at a high level in effluent wastewater [31]. Usually, these substances appear in low μg/L concentration, apart from some places with extreme exposure. However, these xenobiotics appear in high concentrations (mg/L or mg/kg) in extreme cases; for example, in Pakistan, DCF was detected as high as 252–836 μg/L, 695–4968 μg/kg, 125–6632 μg/kg, and 101–257 μg/kg, and IBU 703–1673 μg/L, 2053–6046 μg/kg, 133–1229 μg/kg, and 321–610 μg/kg concentrations in wastewater, sludge, solid waste, and soil, respectively [75]. DCF was detected at a concentration of 2.051 mg/L in Slovakia from untreated urban wastewater [31]. Bibi et al. (2023) reported extreme concentration in wastewater with the highest level at 311,495 µg/L DCF [76]. Metolachlor and terbuthylazine were detected in runoff water at 228.3 μg/L and 290.5 μg/L, respectively. Ibuprofen also was detected at these concentrations in environmental samples and runoff waters [77,78,79]. Additionally, detection data do not necessarily exhaustively represent the concentrations of the active substances in environmental matrices. According to our study, ibuprofen and diclofenac in combination showed synergistic effects at a mixture concentration of 2 mg/L (EC_10_), and terbuthylazine enhanced the cytotoxicity of ibuprofen even at a mixture concentration of 1 mg/L. The quaternary mixture containing carbamazepine, ibuprofen, tebuconazole, and terbuthylazine should also be highlighted, which was proven to be toxic and moderately synergic at even 1 mg/L total concentration, which is below the aforementioned environmental levels. It must be emphasized that the tested concentration ranges in our research were often several orders of magnitude higher than the concentrations of these compounds found in environmental samples. Therefore, in further chronic experiments, testing on other trophic levels should be taken into account. However, one of the main objectives of ecotoxicological studies is to provide data on the toxicity of certain substances using test organisms that enable the estimation of concentration of substances that have no adverse effect on the ecosystem (using assessment factors). Thus, it is crucial not only to test environmentally relevant concentrations but also to gain ecotoxicological information in general, especially considering that sensitive test organisms representing the sensitivity of an ecosystem are not applicable in every case.

The toxicity-enhancing effect of terbuthylazine at low effective levels is foreboding, seeing that it has become nearly a ubiquitous pesticide, particularly in countries where atrazine has been banned. Currently there is no generally accepted all-encompassing explanation for either synergy or one-sided enchantment. In general, the most frequently observed type of synergism occurs when the toxic effect of one substance, referred to as the ‘driver’, is enhanced by a second substance, called the ‘enhancer’. The enhancer can reduce the metabolic inactivation or excretion of the driver, leading to a situation where more of the driver substance is present at the site of effect [80]. On a vertebrate model organism, terbuthylazine and atrazine increased the toxicity of chlorpyrifos, presumably by accelerating its metabolic conversion into a more toxic form, highlighting the possibility of interactions between pesticides when co-occurring in the environment [81]. In a short-term exposure test using a prokaryote as test organism, as applied in this study, the increased toxicity is likely attributed to chemical interactions between terbuthylazine and other substances.

Regarding the toxicology and ecotoxicology of mixtures, there is a significant knowledge gap and inconsistency, coupled with global concern about the assessment, prediction, and impact of pharmaceuticals and pesticide residues in mixtures on the environment.

Alternative statistical analysis methods and a careful selection of chemicals play pivotal roles in achieving a more precise prediction of ecotoxicological effects for environmentally relevant chemicals and the interaction in their cocktails.

## 5. Conclusions

A comprehensive dataset regarding the acute cytotoxicity of three APIs and three pesticides and their all-possible combinations on *Aliivibrio fischeri* was generated. PERMANOVA, uniquely used in this study, was successfully applied to determine the roles of compounds in synergistic, additive, and antagonistic effects in mixtures at different effective concentrations. Diclofenac with ibuprofen, S-metolachlor, and carbamazepine, as well as ibuprofen with S-metolachlor, exhibited synergistic effects in all combinations, even at environmentally relevant concentrations. Diclofenac paired with ibuprofen, carbamazepine, or S-metolachlor induced synergism in the mixtures, parallel to the increase in effective concentrations. Terbuthylazine, now an almost ubiquitous pesticide residue, should also be highlighted, having had no acute cytotoxic effect on *Aliivibrio fischeri* alone, but it significantly enhanced the toxicity of mixtures containing it, especially at low concentrations.

## Figures and Tables

**Table 1:** Acute cytotoxicity of the test chemicals alone and in mixtures. Concentrations are expressed in mg/L resulting in 10, 20, 50, 80, 90, and 95% bioluminescence inhibition in *Aliivibrio fischeri* after 30 min of exposure. 1: carbamazepine, 2: diclofenac, 3: ibuprofen, 4: S-metolachlor, 5: tebuconazole, 6: terbuthylazine. n.t.—non-toxic at the applied concentration. The same table with acronyms instead of number codes can be found in the Supplementary Materials Table S2.

	Effective Concentration Values at Different Effective Sizes												
	EC_10_	EC_20_	EC_50_	EC_80_	EC_90_	EC_95_		EC_10_	EC_20_	EC_50_	EC_80_	EC_90_	EC_95_
	mg/L							mg/L					
1	4	14	86	520	1478	3866	2 + 3 + 4	4	8	22	64	118	208
2	7	13	27	50	77	107	2 + 3 + 5	10	20	54	144	258	442
3	2	5	23	96	222	471	2 + 3 + 6	2	4	14	38	68	114
4	55	100	265	715	n.t.	n.t.	2 + 4 + 5	6	8	20	42	66	98
5	8	14	32	74	120	188	2 + 4 + 6	3	6	22	82	177	360
6	n.t.	n.t.	n.t.	n.t.	n.t.	n.t.	2 + 5 + 6	20	28	51	93	132	182
1 + 2	12	16	28	50	70	94	3 + 4 + 5	2	4	30	178	488	1240
1 + 3	6	12	40	120	230	418	3 + 4 + 6	2	5	33	226	694	1953
1 + 4	16	32	88	232	408	688	3 + 5 + 6	7	15	50	169	345	665
1 + 5	24	32	52	80	108	136	4 + 5 + 6	32	52	114	250	394	598
1 + 6	18	36	120	n.t.	n.t.	n.t.	1 + 2 + 3 + 4	10	14	30	64	96	140
2 + 3	2	4	12	26	40	62	1 + 2 + 3 + 5	12	18	36	72	108	156
2 + 4	10	14	28	56	84	120	1 + 2 + 3 + 6	5	9	28	88	174	324
2 + 5	12	18	34	64	92	126	1 + 2 + 4 + 5	10	16	38	88	144	224
2 + 6	4	10	32	102	202	380	1 + 2 + 4 + 6	6	12	34	99	186	332
3 + 4	6	12	50	184	396	804	1 + 2 + 5 + 6	8	12	24	50	78	116
3 + 5	8	16	56	192	394	760	1 + 3 + 4 + 5	4	12	58	254	598	1312
3 + 6	1	2	18	102	272	672	1 + 3 + 4 + 6	1	4	40	376	1393	4661
4 + 5	12	20	36	64	88	116	1 + 3 + 5 + 6	2	8	48	294	842	2214
4 + 6	30	80	428	2278	6054	14,896	1 + 4 + 5 + 6	2	8	110	1431	6415	25,568
5 + 6	6	16	76	350	850	1918	2 + 3 + 4 + 5	6	12	30	70	114	180
1 + 2 + 3	4	6	12	24	38	54	2 + 3 + 4 + 6	2	6	20	62	124	232
1 + 2 + 4	8	12	28	64	100	154	2 + 3 + 5 + 6	3	6	17	55	107	199
1 + 2 + 5	20	30	54	98	138	190	2 + 4 + 5 + 6	4	8	27	91	186	360
1 + 2 + 6	6	12	28	62	100	154	1 + 2 + 3 + 4 + 5	8	14	38	100	172	284
1 + 3 + 4	2	6	40	228	630	1610	1 + 2 + 3 + 4 + 6	2	6	22	74	150	286
1 + 3 + 5	10	20	66	214	426	802	1 + 2 + 3 + 5 + 6	4	8	22	56	96	158
1 + 3 + 6	2	6	38	218	602	1532	1 + 2 + 4 + 5 + 6	4	9	30	106	220	431
1 + 4 + 5	34	56	126	282	454	702	1 + 3 + 4 + 5 + 6	2	6	42	290	886	2470
1 + 4 + 6	31	55	147	394	699	1187	2 + 3 + 4 + 5 + 6	8	12	22	40	56	78
1 + 5 + 6	20	40	168	684	1548	3288	1 + 2 + 3 + 4 + 5 + 6	2	6	24	100	228	482

**Table 2:** Combination indices for mixtures containing carbamazepine (1), diclofenac (2), ibuprofen (3), S-metolachlor (4), and tebuconazole (5) at effective concentration resulting in 10, 20, 50, 80, 90, and 95% inhibition in bioluminescence in *Aliivibrio fischeri*. CI values were calculated by CompuSyn software using 6 concentration–response data points (EC_10_, EC_20_, EC_50_, EC_80_, EC_90_, and EC_95_). Synergistic (CI < 0.9) and additive effects (0.9 < CI < 1.1) are indicated in bold and italics, respectively. The same table with acronyms instead of number codes can be found in the Supplementary Materials Table S3.

	Combination Index Values at Different Effective Concentrations (Based on the Effective Concentration)					
	EC_10_	EC_20_	EC_50_	EC_80_	EC_90_	EC_95_
1 + 2	2.14	1.30	**0.75**	**0.55**	**0.48**	**0.42**
1 + 3	2.11	1.67	1.17	**0.75**	**0.59**	**0.48**
1 + 4	1.96	1.40	**0.71**	**0.39**	**0.30**	**0.24**
1 + 5	4.17	2.42	1.14	**0.62**	**0.48**	**0.37**
2 + 3	**0.61**	**0.57**	**0.52**	**0.40**	**0.35**	**0.33**
2 + 4	**0.74**	**0.67**	**0.63**	**0.60**	**0.58**	**0.55**
2 + 5	1.51	1.43	1.24	*1.07*	**0.98**	**0.88**
3 + 4	1.48	1.27	1.25	1.11	*1.04*	*1.00*
3 + 5	2.39	2.20	2.18	2.33	2.52	2.77
4 + 5	**0.83**	**0.84**	**0.64**	**0.48**	**0.40**	**0.33**
1 + 2 + 3	1.11	**0.73**	**0.40**	**0.26**	**0.23**	**0.20**
1 + 2 + 4	*1.00*	**0.69**	**0.53**	**0.50**	**0.48**	**0.49**
1 + 2 + 5	3.17	2.36	1.53	1.16	*1.01*	*0.90*
1 + 3 + 4	**0.48**	**0.58**	**0.83**	*1.06*	**1.25**	**1.47**
1 + 3 + 5	2.75	2.35	1.99	1.87	1.91	2.02
1 + 4 + 5	4.14	3.01	2.00	1.59	1.47	1.43
2 + 3 + 4	**0.83**	**0.79**	**0.67**	**0.68**	**0.72**	**0.78**
2 + 3 + 5	2.43	2.40	2.14	2.12	2.22	2.37
2 + 4 + 5	**0.54**	**0.45**	**0.51**	**0.49**	**0.48**	**0.47**
3 + 4 + 5	**0.41**	**0.38**	**0.82**	1.52	2.21	3.20
1 + 2 + 3 + 4	2.13	1.31	**0.78**	**0.54**	**0.45**	**0.40**
1 + 2 + 3 + 5	2.86	1.97	1.18	**0.83**	**0.71**	**0.64**
1 + 2 + 4 + 5	1.24	*0.99*	**0.84**	**0.81**	**0.82**	**0.82**
1 + 3 + 4 + 5	**0.84**	*1.09*	1.36	1.75	2.13	2.62
2 + 3 + 4 + 5	1.12	1.11	*0.92*	**0.80**	**0.76**	**0.74**
1 + 2 + 3 + 4 + 5	1.56	1.25	*1.03*	*0.95*	*0.94*	*0.95*

**Table 3:** Enhancement on cytotoxicity by terbuthylazine at concentrations resulting in 10, 20, 50, 80, 90, and 95% bioluminescence inhibition in *Aliivibrio fischeri* after 30 min of exposure. Enhancement of non-toxic terbuthylazine is expressed as a percent of the required dose change of the other compounds in the mixture not containing terbuthylazine to result in the same effect size as the mixture containing it. n.e.—no enhancing effect. The same table with acronyms instead of number codes can be found in the Supplementary Materials Table S4.

	EC_10_	EC_20_	EC_50_	EC_80_	EC_90_	EC_95_
	Toxicity enhancement by terbuthylazine (%) in the mixtures					
1 + 6	n.e.	n.e.	n.e.	n.e.	n.e.	n.e.
2 + 6	85	81	70	49	34	11
3 + 6	100	90	80	73	69	64
4 + 6	86	80	60	20	n.e.	n.e.
5 + 6	81	71	41	−18	−77	−155
1 + 2 + 6	78	67	56	45	37	27
1 + 3 + 6	85	78	58	19	−16	−63
1 + 4 + 6	14	23	26	25	24	23
1 + 5 + 6	63	44	−44	−280	−537	−975
2 + 3 + 6	56	56	48	35	24	18
2 + 4 + 6	88	81	65	35	6	−33
2 + 5 + 6	27	31	33	36	36	36
3 + 4 + 6	88	82	70	45	22	−8
3 + 5 + 6	61	58	60	61	61	61
4 + 5 + 6	−19	−16	−41	−74	−99	−129
1 + 2 + 3 + 6	30	18	−30	−107	−157	−237
1 + 2 + 4 + 6	58	46	32	13	−5	−21
1 + 2 + 5 + 6	78	78	75	71	68	66
1 + 3 + 4 + 6	69	60	44	7	−24	−63
1 + 3 + 5 + 6	89	78	59	23	−11	−55
1 + 4 + 5 + 6	97	92	51	−185	−695	−1898
2 + 3 + 4 + 6	72	58	49	46	41	37
2 + 3 + 5 + 6	84	85	82	79	77	75
2 + 4 + 5 + 6	64	44	25	−22	−59	−107
3 + 4 + 5 + 6	44	16	33	42	45	48
1 + 2 + 3 + 4 + 6	87	73	53	26	n.e.	−31
1 + 2 + 3 + 5 + 6	79	72	61	50	43	35
1 + 3 + 4 + 5 + 6	68	68	54	27	5	−20
1 + 2 + 4 + 5 + 6	73	66	49	23	2	−23
2 + 3 + 4 + 5 + 6	15	36	53	63	69	72
1 + 2 + 3 + 4 + 5 + 6	83	70	56	31	8	−18

**Table 4:** Permutational multivariate analyses of variance results based on Euclidean distances between chemical combination variations of carbamazepine, diclofenac, ibuprofen, S-metolachlor, and tebuconazole at effect sizes 10%, 20%, 50%, 80%, 90%, and 95% expressed in transformed combination indices. * (*p* = 0.01–0.05), ** (*p* = 0.001–0.01), *** (*p* = 0.0001–0.001).

PERMANOVA (999 Permutations)						
	df	Sum Sq	R^2^	F-Model	*p*-Value	
Tebuconazole	1	3.9289	0.21950	17.0848	0.001	***
Ibuprofen	1	1.9133	0.10689	8.3198	0.009	**
Diclofenac	1	0.9145	0.05109	3.9768	0.033	*
S-Metolachlor	1	1.4574	0.08142	6.3375	0.009	**
Carbamazepine	1	0.8052	0.04498	3.5012	0.056	
Tebuconazole: ibuprofen	1	0.2668	0.01491	1.1603	0.335	
Tebuconazole: diclofenac	1	0.2318	0.01295	1.0082	0.374	
Ibuprofen: diclofenac	1	1.8906	0.10562	8.2211	0.006	**
Tebuconazole: S-metolachlor	1	1.0758	0.06011	4.6783	0.027	*
Tebuconazole: carbamazepine	1	0.1992	0.01113	0.8661	0.394	
S-metolachlor: carbamazepine	1	0.6547	0.03657	2.8468	0.072	
Ibuprofen: S-metolachlor	1	0.1790	0.01000	0.7783	0.464	
Diclofenac: S-metolachlor	1	0.3924	0.02192	1.7064	0.219	
Tebuconazole: ibuprofen: diclofenac	1	0.2695	0.01505	1.1718	0.339	
Tebuconazole: S-metolachlor: carbamazepine	1	1.0217	0.05708	4.4430	0.028	*
Ibuprofen: diclofenac: S-metolachlor	1	0.6288	0.03513	2.7345	0.091	
Residual	9	2.0697	0.11563			
Total	25	17.8992	1.00000			

**Table 5:** Permutational multivariate analyses of variance results based on dynamic time warping distances between the enhancement effect of terbuthylazine on chemical combination variations of carbamazepine, diclofenac, ibuprofen, S-metolachlor, and tebuconazole at effect sizes of 10%, 20%, 50%, 80%, 90%, and 95% expressed as a percentage of enhancement.

	df	Sum Sq	R^2^	F-Model	*p*-Value	
Ibuprofen	1	52.68	0.09762	90.4915	0.002	**
Diclofenac	1	64.30	0.11917	110.4598	0.003	**
Tebuconazole	1	9.42	0.01746	16.1814	0.009	**
S-metolachlor	1	2.21	0.00409	3.7916	0.112	
Carbamazepine	1	36.87	0.06832	63.3309	0.001	***
Ibuprofen: diclofenac	1	74.09	0.13730	127.2731	0.005	**
Ibuprofen: tebuconazole	1	23.52	0.04360	40.4110	0.002	**
Diclofenac: tebuconazole	1	30.66	0.05681	52.6622	0.006	**
Ibuprofen: S-metolachlor	1	5.21	0.00966	8.9548	0.038	*
Diclofenac: S-metolachlor	1	2.38	0.00442	4.0967	0.123	
Tebuconazole: S-metolachlor	1	5.88	0.01090	10.1014	0.013	*
Ibuprofen: carbamazepine	1	11.54	0.02139	19.8274	0.009	**
Diclofenac: carbamazepine	1	38.95	0.07218	66.9076	0.002	**
S-metolachlor: carbamazepine	1	1.23	0.00228	2.1170	0.191	
Ibuprofen: diclofenac: tebuconazole	1	59.17	0.10965	101.6437	0.003	**
Ibuprofen: diclofenac: S-metolachlor	1	3.75	0.00695	6.4437	0.087	.
Ibuprofen: tebuconazole: S-metolachlor	1	0.24	0.00044	0.4069	0.642	
Diclofenac: tebuconazole: S-metolachlor	1	1.81	0.00335	3.1090	0.070	.
Ibuprofen: diclofenac: carbamazepine	1	87.93	0.16295	151.0464	0.001	***
Ibuprofen: S-metolachlor: carbamazepine	1	6.01	0.01114	10.3296	0.033	*
Diclofenac: S-metolachlor: carbamazepine	1	6.97	0.01291	11.9692	0.026	*
Ibuprofen: diclofenac: S-metolachlor: carbamazepine	1	11.29	0.02092	19.3925	0.023	*
Residual	6	3.49	0.00647			
Total	28	539.61	1.00000			

* (*p* = 0.01–0.05), ** (*p* = 0.001–0.01), *** (*p* = 0.0001–0.001). PERMANOVA (999 Permutations).

## Data Availability

Data are available upon request by email to the corresponding author.

## Supplementary Materials

The following supporting information can be downloaded at: https://www.mdpi.com/article/10.3390/toxics12030189/s1, Table S1. Name, CAS number, IUPAC name, structure and LD50 of tested compounds carbamazepine, diclofenac, ibuprofen, S-metolachlor, tebuconazole, terbuthylazine; Table S2. Acute cytotoxicity of the test chemicals alone and in mixtures. Concentrations are expressed in mg/L resulting in 10, 20, 50, 80, 90, and 95% bioluminescence inhibition in *Aliivibrio fischeri* after 30 min of exposure. 1: carbamazepine, 2: diclofenac, 3: ibuprofen, 4: S-metolachlor, 5: tebuconazole, 6: terbuthylazine. N.t.—non-toxic at the applied concentration; Table S3. Combination Indices for mixtures containing carbamazepine (CBZ), diclofenac (DCF), ibuprofen (IBU), S-metolachlor (MTC), and tebuconazole (TBZ) at effective concentration resulting in 10, 20, 50, 80, 90, and 95% inhibition in bioluminescence in *Aliivibrio fischeri*. CI values were calculated by CompuSyn software using 6 concentration-response data points (EC10, EC20, EC50, EC80, EC90, and EC95). Synergistic (CI < 0.9) and additive effects (0.9 < CI < 1.1) are indicated in bold and italics, respectively; Table S4. Enhancement on cytotoxicity by terbuthylazine at concentrations resulting in 10, 20, 50, 80, 90, and 95% bioluminescence inhibition in *Aliivibrio fischeri* after 30 min of exposure. Enhancement of non-toxic terbuthylazine is expressed in percent of the required dose change of the other compounds in the mixture not containing terbuthylazine to result in the same effect size as the mixture containing it. n.e.—no enhancing effect. Carbamazepine (CBZ), diclofenac (DCF), ibuprofen (IBU), S-metolachlor (MTC), and tebuconazole (TBZ), terbuthylazine (TRB); Table S5. Description of synergism or antagonism in drug combination studies analyzed with the CI method described by Chou and Talalay (Chou, 2008) [56]; Table S6. Analyses of multivariate homogeneity: comparing samples containing a specific compound to other samples based on the distance matrix based on transformed combination indices; Table S7. Analyses of multivariate homogeneity: comparing samples containing a specific compound to other samples based on the distance matrix based on transformed combination indices; Table S8. Analyses of multivariate homogeneity: comparing samples containing a specific compound to other samples based on the distance matrix of enhancement by terbuthylazine; Table S9. Analyses of multivariate homogeneity: comparing samples containing compound combinations included in the model of the permutational multivariate analyses of variance to other samples based on the distance matrix of enhancement by Terbuthylazine; Table S10. Analyses of multivariate homogeneity: comparing samples containing compound combinations included in the model of the permutational multivariate analyses of variance to other samples based on the distance matrix of enhancement by terbuthylazine. Table S11. Analyses of multivariate homogeneity: comparing samples containing compound combinations included in the model of the permutational multivariate analyses of variance to other samples based on the distance matrix of enhancement by terbuthylazine. Figure S1. A: Raw CI limits; B: Transformed CI value limit; C: RAW CI data distribution; D: Transformed CI data distribution. Figure S2. Effect level contours and compounds in Non-Metric Multidimensional scaling ordinations based on Euclidean distances of transformed combination indices. Figure S3. Non-Metric Multidimensional scaling ordinations based on Euclidean distances of transformed combination indices by compounds. Figure S4. Non-Metric Multidimensional scaling ordinations based on Euclidean distances of transformed combination indices by compounds. Figure S5. Effect level contours and compounds in Non-Metric Multidimensional scaling ordinations based on Euclidean distances of enhancement by Terbuthylazine. Figure S6. Non-Metric Multidimensional scaling ordinations based on Euclidean distances of enhancement by Terbuthylazine by compounds and compound combinations. Figure S7. Non-Metric Multidimensional scaling ordinations based on Euclidean distances of enhancement by Terbuthylazine by compounds and compound combinations. Figure S8. Weighted mean of transformed Combination Indexes of compound pairs from all the combinations where the chemicals were present in the examined combinations at effect sizes 10%, 20%, 50%, 80%, 90%, and 95%. The strength of synergy (blue) and antagonism (red) is shown by the width of linkage and color intensity.
