# Supplementary material for: The Cocktail Effects on the Acute Cytotoxicity of Pesticides and Pharmaceuticals Frequently Detected in the Environment
Source: Toxics. 2024 Feb 28;12(3):189. doi: 10.3390/toxics12030189 (PMC10974651; doi:10.3390/toxics12030189)
Supplement: Supplementary file 1 [file toxics-12-00189-s001.zip › toxics-2824840-supplementary.pdf]

Supplementary Materials

The Cocktail Effects on the Acute Cytotoxicity of Pesticides and Pharmaceuticals Frequently Detected in the Environment

Balázs Göbölös [1], Rózsa E. Sebők [1], Gyula Szabó [2], Gergő Tóth [1,*], Sándor Szoboszlay [1], Balázs Kriszt [1], Edit Kaszab [1] and Judit Háhn [1]

[1] Department of Environmental Safety, Institute of Aquaculture and Environmental Safety, Hungarian University of Agriculture and Life Sciences, 1 Páter Károly u., 2100 Gödöllő, Hungary; gobolos.balazs@uni-mate.hu (B.G.); rozsa.sebok@gmail.com (R.E.S.); szoboszlay.sandor@uni-mate.hu (S.S.); kriszt.balazs@uni-mate.hu (B.K.); kaszab.edit@uni-mate.hu (E.K.); hahn.judit@uni-mate.hu (J.H.)
[2] Department of Environmental Toxicology, Institute of Aquaculture and Environmental Safety, Hungarian University of Agriculture and Life Sciences, 1 Páter Károly u., 2100 Gödöllő, Hungary; gyula.szabo.mate@gmail.com
* Correspondence: toth.gergo@uni-mate.hu

Supplementary Tables

Table S1. Name, CAS number, IUPAC name, structure and LD_{50} of tested compounds carbamazepine, diclofenac, ibuprofen, S-metolachlor, tebuconazole, terbuthylazine.

Name	CAS Number	IUPAC Name	Structure	Oral LD_{50} Rat mg/kg	References
Carbamazepine	298-46-4	benzo[b][1] benzazepine-11-carboxamide	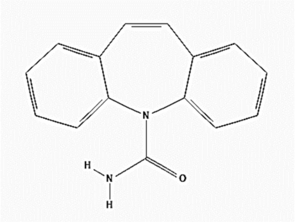	1957	National Center for Biotechnology Information (2024). PubChem Compound Summary for CID 2554, Carbamazepine. Retrieved 17 January 2024 from https://pubchem.ncbi.nlm.nih.gov/compound/Carbamazepine (accessed on 23 January 2024).
Diclofenac	15307-86-5	2-[2-(2,6-dichloroanilino) phenyl] acetic acid	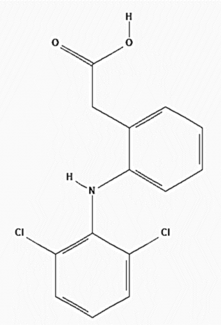	62.5	National Center for Biotechnology Information (2024). PubChem Compound Summary for CID 3033, Diclofenac. Retrieved January 17, 2024 from https://pubchem.ncbi.nlm.nih.gov/compound/Diclofenac (accessed on 23 January 2024).

Ibuprofen	15687-27-1	2-[4-(2-methylpropyl) phenyl]propanoic acid	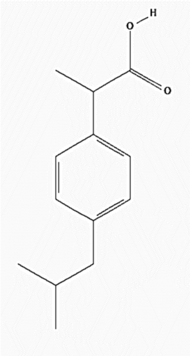	636	National Center for Biotechnology Information (2024). PubChem Compound Summary for CID 3672, Ibuprofen. Retrieved January 17, 2024 from https://pubchem.ncbi.nlm.nih.gov/compound/Ibuprofen (accessed on 23 January 2024).
S-metolachlor	87392-12-9	2-chloro-N-(2-ethyl-6-methylphenyl)-N-[(2S)-1-methoxypropan-2-yl]acetamide	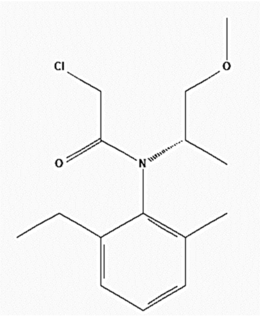	2780	National Center for Biotechnology Information (2024). PubChem Compound Summary for CID 11140605, S-Metolachlor. Retrieved January 17, 2024 from https://pubchem.ncbi.nlm.nih.gov/compound/S-Metolachlor (accessed on 23 January 2024). United States Environmental Protection Agency (2024). Retrived January 17, 2024 from https://www3.epa.gov/pesticides/endanger/litstatus/effects/redleg-frog/2010/metolachlor-s/appendix-j1.pdf (accessed on 23 January 2024).
Tebuconazole	107534-96-3	1-(4-chlorophenyl)-4,4-dimethyl-3-(1,2,4-triazol-1-ylmethyl)pentan-3-ol	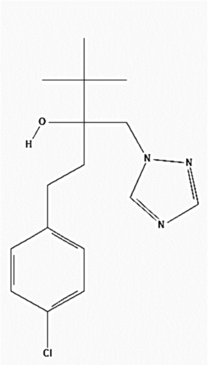	3352	National Center for Biotechnology Information (2024). PubChem Compound Summary for CID 86102, Tebuconazole. Retrieved January 17, 2024 from https://pubchem.ncbi.nlm.nih.gov/compound/Tebuconazole (accessed on 23 January 2024).
Terbuthylazine	5915-41-3	2-N-tert-butyl-6-chloro-4-N-ethyl-1,3,5-triazine-2,4-diamine	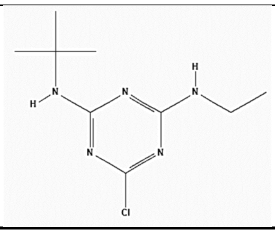	1845	National Center for Biotechnology Information (2024). PubChem Compound Summary for CID 22206, Terbuthylazine. Retrieved January 17, 2024

	from https://pubchem.ncbi.nlm.nih.gov/compound/Terbuthylazine (accessed on 23 January 2024).

Table S2. Acute cytotoxicity of the test chemicals alone and in mixtures. Concentrations are expressed in mg/L resulting in 10, 20, 50, 80, 90, and 95% bioluminescence inhibition in *Aliivibrio fischeri* after 30 minutes of exposure. 1: carbamazepine, 2: diclofenac, 3: ibuprofen, 4: S-metolachlor, 5: tebuconazole, 6: terbuthylazine. N.t. – non-toxic at the applied concentration.

	EC_{10}	EC_{20}	EC_{50}	EC_{80}	EC_{90}	EC_{95}
			mg/L			
CBZ	4	14	86	520	1478	3866
DCF	7	13	27	50	77	107
IBU	2	5	23	96	222	471
MTC	55	100	265	715	n.t.	n.t.
TBZ	8	14	32	74	120	188
TRB	n.t.	n.t.	n.t.	n.t.	n.t.	n.t.
CBZ+DCF	12	16	28	50	70	94
CBZ+IBU	6	12	40	120	230	418
CBZ+MTC	16	32	88	232	408	688
CBZ+TBZ	24	32	52	80	108	136
CBZ+TRB	18	36	120	n.t.	n.t.	n.t.
DCF+IBU	2	4	12	26	40	62
DCF+MTC	10	14	28	56	84	120
DCF+TBZ	12	18	34	64	92	126
DCF+TRB	4	10	32	102	202	380
IBU+MTC	6	12	50	184	396	804
IBU+TBZ	8	16	56	192	394	760
IBU+TRB	1	2	18	102	272	672
MTC+TBZ	12	20	36	64	88	116
MTC+TRB	30	80	428	2278	6054	14896
TBZ+TRB	6	16	76	350	850	1918
CBZ+DCF+IBU	4	6	12	24	38	54
CBZ+DCF+MTC	8	12	28	64	100	154
CBZ+DCF+TBZ	20	30	54	98	138	190
CBZ+DCF+TRB	6	12	28	62	100	154
CBZ+IBU+MTC	2	6	40	228	630	1610
CBZ+IBU+TBZ	10	20	66	214	426	802
CBZ+IBU+TRB	2	6	38	218	602	1532
CBZ+MTC+TBZ	34	56	126	282	454	702
CBZ+MTC+TRB	31	55	147	394	699	1187
CBZ+TBZ+TRB	20	40	168	684	1548	3288
DCF+IBU+MTC	4	8	22	64	118	208
DCF+IBU+TBZ	10	20	54	144	258	442
DCF+IBU+TRB	2	4	14	38	68	114
DCF+MTC+TBZ	6	8	20	42	66	98
DCF+MTC+TRB	3	6	22	82	177	360

DCF+TBZ+TRB	20	28	51	93	132	182
IBU+MTC+TBZ	2	4	30	178	488	1240
IBU+MTC+TRB	2	5	33	226	694	1953
IBU+TBZ+TRB	7	15	50	169	345	665
MTC+TBZ+TRB	32	52	114	250	394	598
CBZ+DCF+IBU+MTC	10	14	30	64	96	140
CBZ+DCF+IBU+TBZ	12	18	36	72	108	156
CBZ+DCF+IBU+TRB	5	9	28	88	174	324
CBZ+DCF+MTC+TBZ	10	16	38	88	144	224
CBZ+DCF+MTC+TRB	6	12	34	99	186	332
CBZ+DCF+TBZ+TRB	8	12	24	50	78	116
CBZ+IBU+MTC+TBZ	4	12	58	254	598	1312
CBZ+IBU+MTC+TRB	1	4	40	376	1393	4661
CBZ+IBU+TBZ+TRB	2	8	48	294	842	2214
CBZ+MTC+TBZ+TRB	2	8	110	1431	6415	25568
DCF+IBU+MTC+TBZ	6	12	30	70	114	180
DCF+IBU+MTC+TRB	2	6	20	62	124	232
DCF+IBU+TBZ+TRB	3	6	17	55	107	199
DCF+MTC+TBZ+TRB	4	8	27	91	186	360
CBZ+DCF+IBU+MTC+TBZ	8	14	38	100	172	284
CBZ+DCF+IBU+MTC+TRB	2	6	22	74	150	286
CBZ+DCF+IBU+TBZ+TRB	4	8	22	56	96	158
CBZ+DCF+MTC+TBZ+TRB	4	9	30	106	220	431
CBZ+IBU+MTC+TBZ+TRB	2	6	42	290	886	2470
DCF+IBU+MTC+TBZ+TRB	8	12	22	40	56	78
CBZ+DCF+IBU+MTC+TBZ+TRB	2	6	24	100	228	482

Table S3. Combination Indices for mixtures containing carbamazepine (CBZ), diclofenac (DCF), ibuprofen (IBU), S-metolachlor (MTC), and tebuconazole (TBZ) at effective concentration resulting in 10, 20, 50, 80, 90, and 95% inhibition in bioluminescence in *Aliivibrio fischeri*. CI values were calculated by CompuSyn software using 6 concentration-response data points (EC_{10}, EC_{20}, EC_{50}, EC_{80}, EC_{90}, and EC_{95}). Synergistic (CI < 0.9) and additive effects (0.9 < CI < 1.1) are indicated in bold and italics, respectively.

	**Combination Index Values at Effective Concentrations**					
	**EC_{10}**	**EC_{20}**	**EC_{50}**	**EC_{80}**	**EC_{90}**	**EC_{95}**
CBZ+DCF	2.142	1.301	**0.749**	**0.546**	**0.478**	**0.424**
CBZ+IBU	2.110	1.673	1.172	**0.755**	**0.594**	**0.477**
CBZ+MTC	1.956	1.398	**0.707**	**0.389**	**0.296**	**0.243**
CBZ+TBZ	4.167	2.416	1.143	**0.622**	**0.484**	**0.374**
DCF+IBU	**0.607**	**0.574**	**0.525**	**0.397**	**0.349**	**0.335**
DCF+MTC	**0.743**	**0.668**	**0.629**	**0.596**	**0.578**	**0.554**
DCF+TBZ	1.508	1.431	1.238	*1.071*	**0.977**	**0.884**
IBU+MTC	1.485	1.271	1.252	1.107	*1.045*	*1.003*
IBU+TBZ	2.391	2.203	2.183	2.326	2.520	2.772
MTC+TBZ	**0.833**	**0.838**	**0.637**	**0.480**	**0.399**	**0.332**
CBZ+DCF+IBU	1.112	**0.728**	**0.399**	**0.260**	**0.230**	**0.199**
CBZ+DCF+MTC	*1.000*	**0.691**	**0.534**	**0.496**	**0.481**	**0.486**
CBZ+DCF+TBZ	3.186	2.363	1.532	1.157	*1.009*	*0.904*
CBZ+IBU+MTC	**0.481**	**0.578**	**0.831**	*1.063*	1.249	1.472
CBZ+IBU+TBZ	2.747	2.351	1.986	1.868	1.911	2.016
CBZ+MTC+TBZ	4.140	3.007	2.004	1.593	1.475	1.432

DCF+IBU+MTC	**0.834**	**0.792**	**0.669**	**0.681**	**0.718**	**0.781**
DCF+IBU+TBZ	2.428	2.404	2.144	2.117	2.216	2.366
DCF+MTC+TBZ	**0.539**	**0.451**	**0.511**	**0.488**	**0.485**	**0.473**
IBU+MTC+TBZ	**0.410**	**0.381**	**0.817**	1.520	2.208	3.205
CBZ+DCF+IBU+MTC	2.130	1.310	**0.777**	**0.542**	**0.454**	**0.403**
CBZ+DCF+IBU+TBZ	2.864	1.971	1.183	**0.829**	**0.714**	**0.636**
CBZ+DCF+MTC+TBZ	1.240	*0.986*	**0.844**	**0.810**	**0.818**	**0.825**
CBZ+IBU+MTC+TBZ	**0.842**	*1.088*	1.363	1.752	2.129	2.624
DCF+IBU+MTC+TBZ	1.119	1.112	*0.922*	**0.796**	**0.757**	**0.743**
CBZ+DCF+IBU+MTC+TBZ	1.556	1.254	*1.027*	*0.949*	*0.936*	*0.952*

Table S4. Enhancement on cytotoxicity by terbuthylazine at concentrations resulting in 10, 20, 50, 80, 90, and 95% bioluminescence inhibition in *Aliivibrio fischeri* after 30 minutes of exposure. Enhancement of non-toxic terbuthylazine is expressed in percent of the required dose change of the other compounds in the mixture not containing terbuthylazine to result in the same effect size as the mixture containing it. n.e. – no enhancing effect. Carbamazepine (CBZ), diclofenac (DCF), ibuprofen (IBU), S-metolachlor (MTC), and tebuconazole (TBZ), terbuthylazine (TRB).

	**EC_{10}**	**EC_{20}**	**EC_{50}**	**EC_{80}**	**EC_{90}**	**EC_{95}**
	**Toxicity Enhancement by Terbuthylazine (%)**					
CBZ+TRB	n.e.	n.e.	n.e.	n.e.	n.e.	n.e.
DCF+TRB	85	81	70	49	34	11
IBU+TRB	100	90	80	73	69	64
MTC+TRB	86	80	60	20	n.e.	n.e.
TBZ+TRB	81	71	41	-18	-77	-155
CBZ+DCF+TRB	78	67	56	45	37	27
CBZ+IBU+TRB	85	78	58	19	-16	-63
CBZ+MTC+TRB	14	23	26	25	24	23
CBZ+TBZ+TRB	63	44	-44	-280	-537	-975
DCF+IBU+TRB	56	56	48	35	24	18
DCF+MTC+TRB	88	81	65	35	6	-33
DCF+TBZ+TRB	27	31	33	36	36	36
IBU+MTC+TRB	88	82	70	45	22	-8
IBU+TBZ+TRB	61	58	60	61	61	61
MTC+TBZ+TRB	-19	-16	-41	-74	-99	-129
CBZ+DCF+IBU+TRB	30	18	-30	-107	-157	-237
CBZ+DCF+MTC+TRB	58	46	32	13	-5	-21
CBZ+DCF+TBZ+TRB	78	78	75	71	68	66
CBZ+IBU+MTC+TRB	69	60	44	7	-24	-63
CBZ+IBU+TBZ+TRB	89	78	59	23	-11	-55
CBZ+MTC+TBZ+TRB	97	92	51	-185	-695	-1898
DCF+IBU+MTC+TRB	72	58	49	46	41	37
DCF+IBU+TBZ+TRB	84	85	82	79	77	75
DCF+MTC+TBZ+TRB	64	44	25	-22	-59	-107
IBU+MTC+TBZ+TRB	44	16	33	42	45	48
CBZ+DCF+IBU+MTC+TRB	87	73	53	26	n.e.	-31
CBZ+DCF+IBU+TBZ+TRB	79	72	61	50	43	35
CBZ+IBU+MTC+TBZ+TRB	68	68	54	27	5	-20
CBZ+DCF+MTC+TBZ+TRB	73	66	49	23	2	-23
DCF+IBU+MTC+TBZ+TRB	15	36	53	63	69	72
CBZ+DCF+IBU+MTC+TBZ+TRB	83	70	56	31	8	-18

Table S5. Description of synergism or antagonism in drug combination studies analyzed with the CI method described by Chou and Talalay (Chou, 2008).

CI Lower Limit	CI Upper Limit	Transformed CI Lower Limit	Transformed CI Upper Limit	Description
	<0.1	>0.9		very strong synergism
0.1	0.3	0.7	0.9	strong synergism
0.3	0.7	0.3	0.7	synergism
0.7	0.85	0.15	0.3	moderate synergism
0.85	0.9	0.1	0.15	slight synergism
0.9	1.1	-0.04139269	0.1	nearly additive
1.1	1.2	-0.07918125	-0.04139269	slight antagonism
1.2	1.45	-0.16136800	-0.07918125	moderate antagonism
1.45	3.3	-0.51851394	-0.16136800	antagonism
3.3	10	-1	-0.51851394	strong antagonism
>10			<-1	very strong antagonism

Table S6. Analyses of multivariate homogeneity: comparing samples containing a specific compound to other samples based on the distance matrix based on transformed combination indices.

**Tebuconazole (permutations = 999)**					
**Average distance to median:**		Not containing	Containing		
		0.6118	0.7249		
**Analyses of variance**	Df	Sum Sq	Mean Sq	F value	Pr(>F)
Groups	1	0.0811	0.081054	0.6015	0.446
Residuals	24	3.2339	0.134745		
**S-Metolachlor (permutations = 999)**					
**Average distance to median:**		Not containing	Containing		
		0.785	0.708		
**Analyses of variance**	Df	Sum Sq	Mean Sq	F value	Pr(>F)
Groups	1	0.0376	0.037587	0.2384	0.64
Residuals	24	3.7838	0.157659		
**Ibuprofen (permutations = 999)**					
**Average distance to median:**		Not containing	Containing		
		0.6994	0.7985		
**Analyses of variance**	Df	Sum Sq	Mean Sq	F value	Pr(>F)
Groups	1	0.0624	0.062396	0.4688	0.516
Residuals	24	3.1941	0.133087		
**Diclofenac (permutations = 999)**					
**Average distance to median:**		Not containing	Containing		
		0.8845	0.6482		
**Analyses of variance**	Df	Sum Sq	Mean Sq	F value	Pr(>F)
Groups	1	0.35427	0.35427	2.7887	0.114
Residuals	24	3.04891	0.12704		
**Carbamazepine (permutations = 999)**					
**Average distance to median:**		Not containing	Containing		
		0.806	0.727		
**Analyses of variance**	Df	Sum Sq	Mean Sq	F value	Pr(>F)
Groups	1	0.0396	0.039577	0.2759	0.607
Residuals	24	3.4426	0.143442		

Table S7. Analyses of multivariate homogeneity: comparing samples containing compound combinations included in the model of the permutational multivariate analyses of variance to other samples based on the distance matrix based on transformed combination indices.

**Ibuprofen and Diclofenac (permutations = 999)**					
**Average distance to median:**		Not containing	Containing		
		0.8181	0.7003		
**Analyses of variance**	Df	Sum Sq	Mean Sq	F value	Pr(>F)
Groups	1	0.07684	0.076843	0.6143	0.466
Residuals	24	3.00217	0.125090		
**Tebuconazole and Carbamazepine (permutations = 999)**					
**Average distance to median:**		Not containing	Containing		
		0.7694	0.5779		
**Analyses of variance**	Df	Sum Sq	Mean Sq	F value	Pr(>F)
Groups	1	0.2030	0.20295	1.5106	0.243
Residuals	24	3.2245	0.13435		
**S-Metolachlor and Carbamazepine (permutations = 999)**					
**Average distance to median:**		Not containing	Containing		
		0.8009	0.7434		
**Analyses of variance**	Df	Sum Sq	Mean Sq	F value	Pr(>F)
Groups	1	0.0183	0.018285	0.1273	0.745
Residuals	24	3.4462	0.143592		
**Tebuconazole and S-Metolachlor (permutations = 999)**					
**Average distance to median:**		Not containing	Containing		
		0.7807	0.7494		
**Analyses of variance**	Df	Sum Sq	Mean Sq	F value	Pr(>F)
Groups	1	0.0054	0.005397	0.0363	0.858
Residuals	24	3.5653	0.148553		
**Tebuconazole, S-Metolachlor and Carbamazepine (permutations = 999)**					
**Average distance to median:**		Not containing	Containing		
		0.7929	0.5323		
**Analyses of variance**	Df	Sum Sq	Mean Sq	F value	Pr(>F)
Groups	1	0.2297	0.22971	1.7333	0.196
Residuals	24	3.1806	0.13253		

Table S8. Analyses of multivariate homogeneity: comparing samples containing a specific compound to other samples based on the distance matrix of enhancement by terbuthylazine.

**Ibuprofen (permutations = 999)**					
**Average distance to median:**		Not containing	Containing		
		3.3240	0.8372		
**Analyses of variance**	Df	Sum Sq	Mean Sq	F value	Pr(>F)
Groups	1	44.35	44.355	2.6307	0.094
Residuals	27	455.24	16.861		
**Diclofenac (permutations = 999)**					
**Average distance to median:**		Not containing	Containing		
		3.3188	0.8077		
**Analyses of variance**	Df	Sum Sq	Mean Sq	F value	Pr(>F)
Groups	1	45.23	45.225	2.757	0.079
Residuals	27	442.91	16.404		
**Tebuconazole (permutations = 999)**					
**Average distance to median:**		Not containing	Containing		
		0.7896	2.9089		
**Analyses of variance**	Df	Sum Sq	Mean Sq	F value	Pr(>F)
Groups	1	32.22	32.215	1.8659	0.211
Residuals	27	466.16	17.265		
**S-Metolachlor (permutations = 999)**					
**Average distance to median:**		Not containing	Containing		
		1.782	2.053		
**Analyses of variance**	Df	Sum Sq	Mean Sq	F value	Pr(>F)
Groups	1	0.53	0.5331	0.0278	0.894
Residuals	27	517.84	19.1793		
**Carbamazepine (permutations = 999)**					
**Average distance to median:**		Not containing	Containing		
		0.9497	2.7849		
**Analyses of variance**	Df	Sum Sq	Mean Sq	F value	Pr(>F)
Groups	1	24.39	24.390	1.382	0.322
Residuals	27	476.51	17.648		

Table S9. Analyses of multivariate homogeneity: comparing samples containing compound combinations included in the model of the permutational multivariate analyses of variance to other samples based on the distance matrix of enhancement by Terbuthylazine.

**Ibuprofen & Diclofenac (permutations = 999)**					
**Average distance to median:**		Not containing	Containing		
		2.2907	0.9689		
**Analyses of variance**	Df	Sum Sq	Mean Sq	F value	Pr(>F)
Groups	1	44481	10.121	0.5679	0.569
Residuals	27	481.17	17.821		
**Ibuprofen & Tebuconazole (permutations = 999)**					
**Average distance to median:**		Not containing	Containing		
		2.3865 0.6764			
**Analyses of variance**	Df	Sum Sq	Mean Sq	F value	Pr(>F)
Groups	1	16.94	16.941	0.9744	0.343
Residuals	27	469.44	17.387		
**Diclofenac & Tebuconazole (permutations = 999)**					
**Average distance to median:**		Not containing	Containing		
		2.3508	0.8088		
**Analyses of variance**	Df	Sum Sq	Mean Sq	F value	Pr(>F)
Groups	1	13.77	13.775	0.7842	0.423
Residuals	27	474.28	17.566		
**Ibuprofen & S-Metolachlor (permutations = 999)**					
**Average distance to median:**		Not containing	Containing		
		2.4727	0.5758		
**Analyses of variance**	Df	Sum Sq	Mean Sq	F value	Pr(>F)
Groups	1	20.84	20.844	1.2016	0.248
Residuals	27	468.37	17.347		
**Diclofenac & S-Metolachlor (permutations = 999)**					
**Average distance to median:**		Not containing	Containing		
		2.4620	0.5749		
**Analyses of variance**	Df	Sum Sq	Mean Sq	F value	Pr(>F)
Groups	1	20.63	20.631	1.1483	0.274
Residuals	27	485.10	17.967		
**Tebuconazole & S-Metolachlor (permutations = 999)**					
**Average distance to median:**		Not containing	Containing		
		1.376	3.514		
**Analyses of variance**	Df	Sum Sq	Mean Sq	F value	Pr(>F)
Groups	1	26.47	26.471	1.4179	0.189
Residuals	27	504.08	18.670		
**Ibuprofen & Carbamazepine (permutations = 999)**					
**Average distance to median:**		Not containing	Containing		
		2.3475	0.7299		
**Analyses of variance**	Df	Sum Sq	Mean Sq	F value	Pr(>F)
Groups	1	15.16	15.159	0.8138	0.448
Residuals	27	502.96	18.628		

Table S10. Analyses of multivariate homogeneity: comparing samples containing compound combinations included in the model of the permutational multivariate analyses of variance to other samples based on the distance matrix of enhancement by terbuthylazine.

**Diclofenac & Carbamazepine (permutations = 999)**					
**Average distance to median:**		Not containing	Containing		
		2.370	0.854		
**Analyses of variance**	Df	Sum Sq	Mean Sq	F value	Pr(>F)
Groups	1	13.32	13.316	0.7481	0.479
Residuals	27	480.58	17.799		
**S-Metolachlor & Carbamazepine (permutations = 999)**					
**Average distance to median:**		Not containing	Containing		
		1.534	2.980		
**Analyses of variance**	Df	Sum Sq	Mean Sq	F value	Pr(>F)
Groups	1	44543	12.127	0.6212	0.545
Residuals	27	527.12	19.523		
**Ibuprofen & Diclofenac & Tebuconazole (permutations = 999)**					
**Average distance to median:**		Not containing	Containing		
		2.1041	0.6308		
**Analyses of variance**	Df	Sum Sq	Mean Sq	F value	Pr(>F)
Groups	1	7.48	7.4845	0.4229	0.474
Residuals	27	477.90	17.6999		
**Ibuprofen & Diclofenac & S-Metolachlor (permutations = 999)**					
**Average distance to median:**		Not containing	Containing		
		2.1515	0.6405		
**Analyses of variance**	Df	Sum Sq	Mean Sq	F value	Pr(>F)
Groups	1	7.87	7.8727	0.4446	0.439
Residuals	27	478.10	17.7075		
**Ibuprofen & Tebuconazole & S-Metolachlor (permutations = 999)**					
**Average distance to median:**		Not containing	Containing		
		2.1457	0.6966		
**Analyses of variance**	Df	Sum Sq	Mean Sq	F value	Pr(>F)
Groups	1	44401	7.2414	0.4099	0.487
Residuals	27	476.95	17.6648		
**Diclofenac & Tebuconazole & S-Metolachlor (permutations = 999)**					
**Average distance to median:**		Not containing	Containing		
		2.1302	0.7897		
**Analyses of variance**	Df	Sum Sq	Mean Sq	F value	Pr(>F)
Groups	1	44367	6.196	0.3431	0.57
Residuals	27	487.57	18.058		
**Ibuprofen & Diclofenac & Carbamazepine (permutations = 999)**					
**Average distance to median:**		Not containing	Containing		
		2.071	1.185		
**Analyses of variance**	Df	Sum Sq	Mean Sq	F value	Pr(>F)
Groups	1	2.71	2.7079	0.1487	0.872
Residuals	27	491.63	18.2084		

Table S11. Analyses of multivariate homogeneity: comparing samples containing compound combinations included in the model of the permutational multivariate analyses of variance to other samples based on the distance matrix of enhancement by terbuthylazine.

**Ibuprofen & S-Metolachlor & Carbamazepine (permutations = 999)**					
**Average distance to median:**		Not containing	Containing		
		2.1908	0.2503		
**Analyses of variance**	Df	Sum Sq	Mean Sq	F value	Pr(>F)
Groups	1	12.99	12.986	0.7132	0.239
Residuals	27	491.58	18.207		
**Diclofenac & S-Metolachlor & Carbamazepine (permutations = 999)**					
**Average distance to median:**		Not containing	Containing		
		2.2024	0.1945		
**Analyses of variance**	Df	Sum Sq	Mean Sq	F value	Pr(>F)
Groups	1	13.90	13.903	0.7676	0.211
Residuals	27	489.06	18.113		
**Ibuprofen & Diclofenac & S-Metolachlor & Carbamazepine (permutations = 999)**					
**Average distance to median:**		Not containing	Containing		
		2.0618	0.1195		
**Analyses of variance**	Df	Sum Sq	Mean Sq	F value	Pr(>F)
Groups	1	44379	7.0248	0.3913	0.155
Residuals	27	484.75	17.9536		

Supplementary Figures

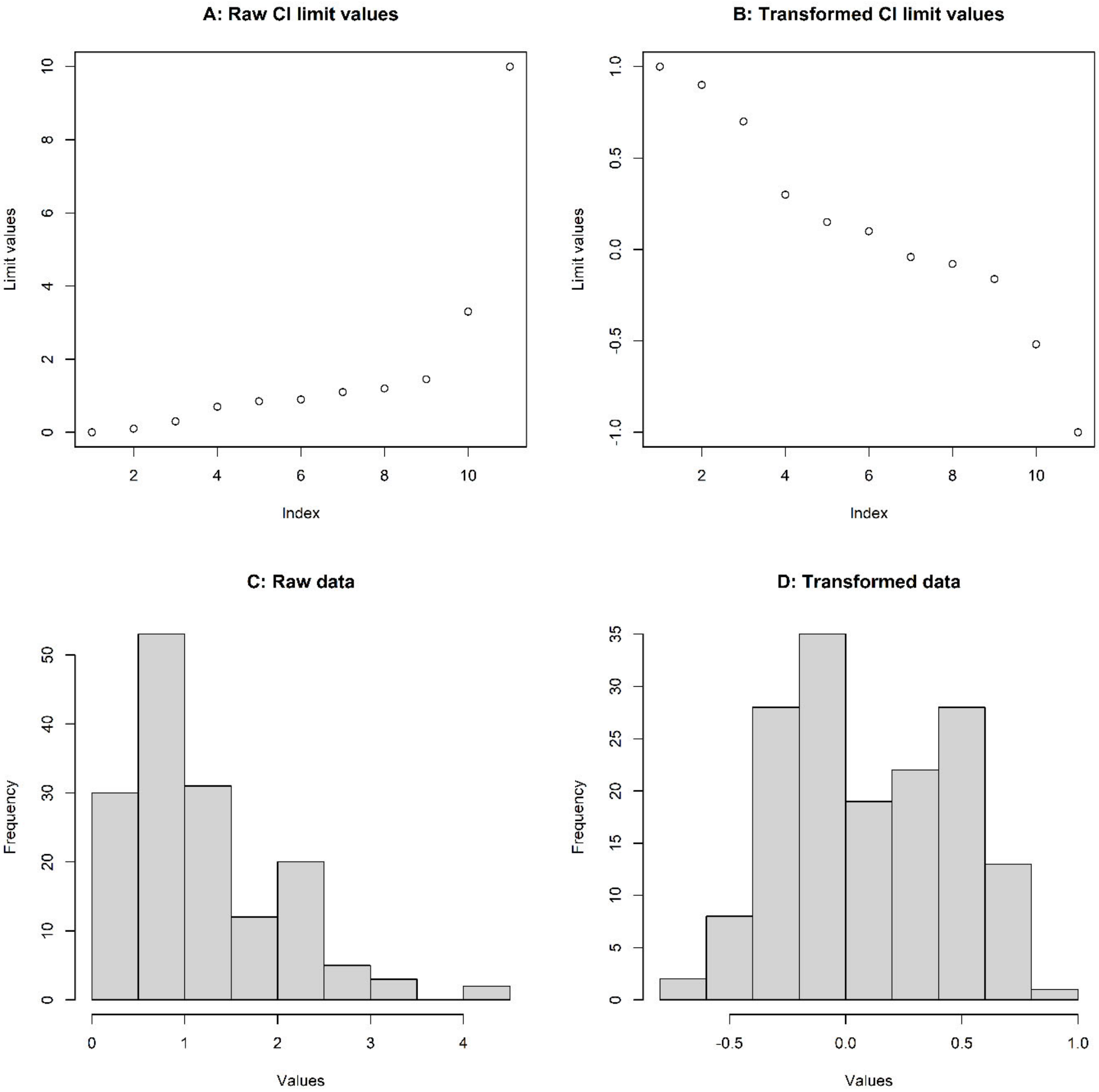

Figure S1. (**A**) Raw CI limits; (**B**) Transformed CI value limit; (**C**) RAW CI data distribution; (**D)** Transformed CI data distribution.

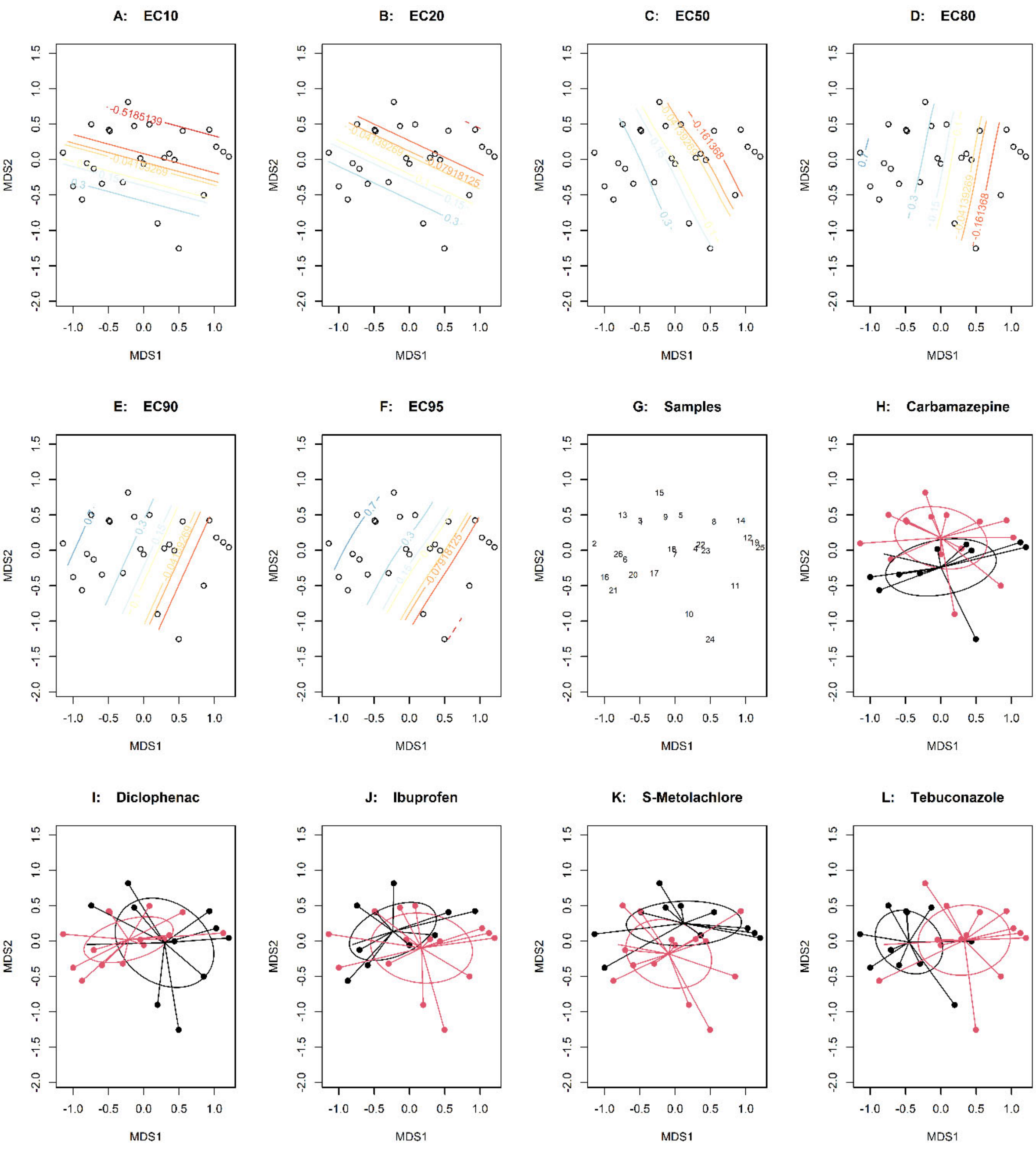

Figure S2. Effect level contours and compounds in Non-Metric Multidimensional scaling ordinations based on Euclidean distances of transformed combination indices. (**A**) Contours of transformed combination index values at 10% effect level. Strength of synergy (blue) and antagonism (red) is shown by color and intensity; (**B**) Contours of transformed combination index values at 20% effect level. Strength of synergy (blue) and antagonism (red) is shown by color and intensity; (**C**) Contours of transformed combination index values at 50% effect level**.** Strength of synergy (blue) and antagonism (red) is shown by color and intensity; (**D**) Contours of transformed combination index values at 80% effect level**.** Strength of synergy (blue) and antagonism (red) is shown by color and intensity; (**E**) Contours of transformed combination index values at 90% effect level. Strength of synergy (blue) and antagonism (red) is shown by color and intensity; (**F**) Contours of transformed combination index values at 95% effect level**.** Strength of synergy (blue) and antagonism (red) is shown by color and intensity ; (**G**) Samples. 1: Carbamazepine + Diclofenac; 2: Carbamazepine + Diclofenac + Ibuprofen; 3: Carbamazepine + Diclofenac + Ibuprofen + S-metolachlor; 4: Carbamazepine + Diclofenac + Ibuprofen + S-metolachlor + Tebuconazole; 5: Carbamazepine + Diclofenac + Ibuprofen + Tebuconazole; 6: Carbamazepine + Diclofenac + S-metolachlor; 7: Carbamazepine + Diclofenac + S-metolachlor + Tebuconazole; 8: Carbamazepine + Diclofenac + Tebuconazole; 9: Carbamazepine + Ibuprofen**;** 10: Carbamazepine + Ibuprofen + S-metolachlor; 11: Carbamazepine + Ibu-

profen + S-metolachlor + Tebuconazole; 12: Carbamazepine + Ibuprofen + Tebuconazole; 13: Carbamazepine + S-metolachlor; 14: Carbamazepine + S-metolachlor + Tebuconazole; 15: Carbamazepine + Tebuconazole; 16: Diclofenac + Ibuprofen; 17: Diclofenac + Ibuprofen + S-metolachlor; 18: Diclofenac + Ibuprofen + S-metolachlor + Tebuconazole; 19: Diclofenac + Ibuprofen + Tebuconazole; 20: Diclofenac + S-metolachlor; 21: Diclofenac + S-metolachlor + Tebuconazole; 22: Diclofenac +Tebuconazole; 23: Ibuprofen + S-metolachlor; 24: Ibuprofen + S-metolachlor + Tebuconazole; 25: Ibuprofen + Tebuconazole; 26: S-metolachlor + Tebuconazole; (**H**) Carbamazepine; (**I**) Diclofenac; (**J**) Ibuprofen; (**K**) S-metolachlor; (**L**): Tebuconazole.

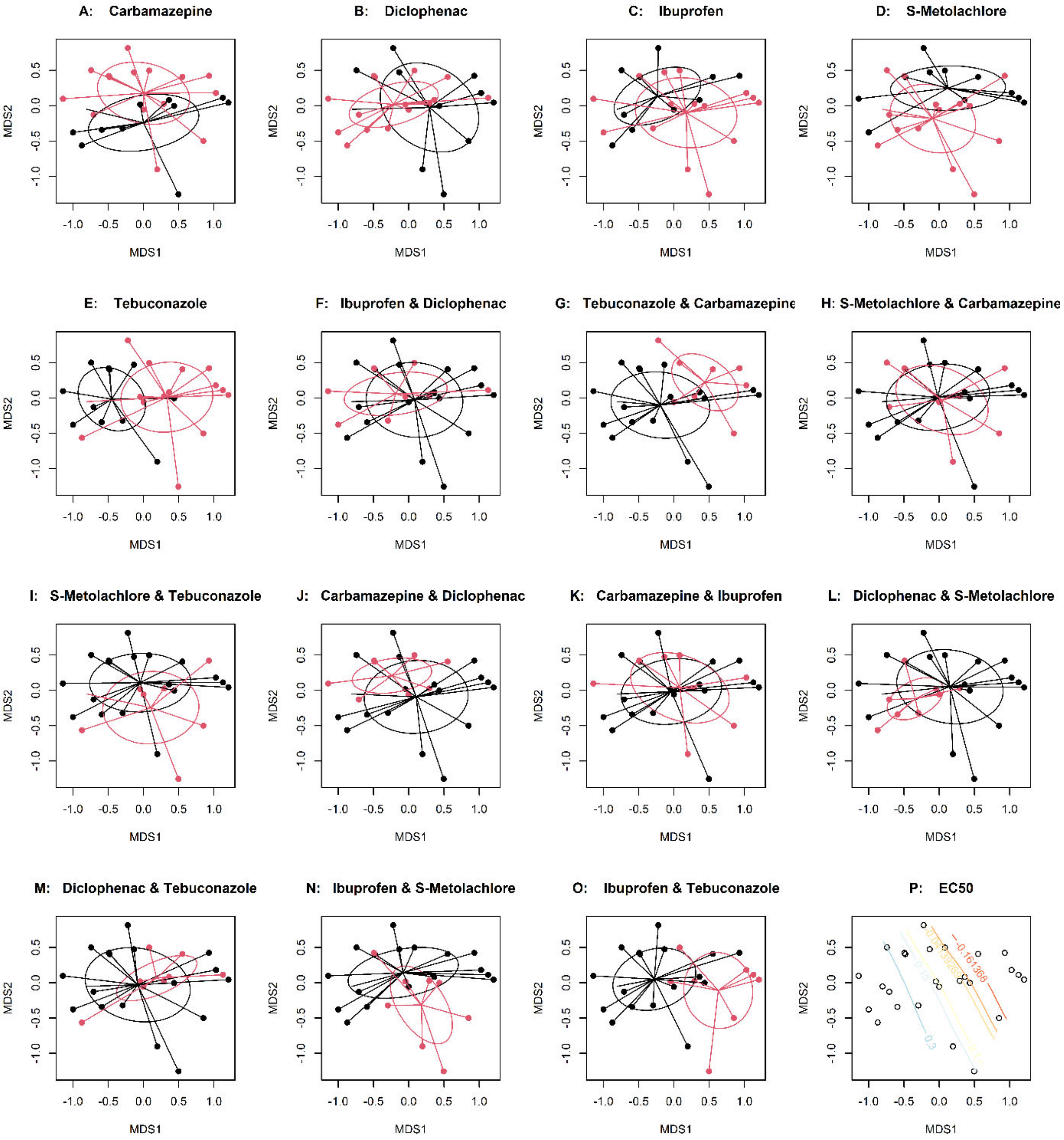

Figure S3. Non-Metric Multidimensional scaling ordinations based on Euclidean distances of transformed combination indices by compounds. (A) Carbamazepine; (B) Diclofenac; (C) Ibuprofen; (D) S-Metolachlor; (E) Tebuconazole; (F) Ibuprofen & Diclofenac; (G) Tebuconazole & Carbamazepine; (H) S-Metolachlor & Carbamazepine; (I) S-Metolachlor & Tebuconazole; (J) Carbamazepine & Diclofenac; (K) Carbamazepine & Ibuprofen; (L) Diclofenac & S-Metolachlor; (M) Diclofenac & Tebuconazole; (N) Ibuprofen & S-Metolachlor; (O) Ibuprofen & Tebuconazole; (P) Contours of transformed combination index values at 50% effect level. Strength of synergy (blue) and antagonism (red) is shown by color and intensity.

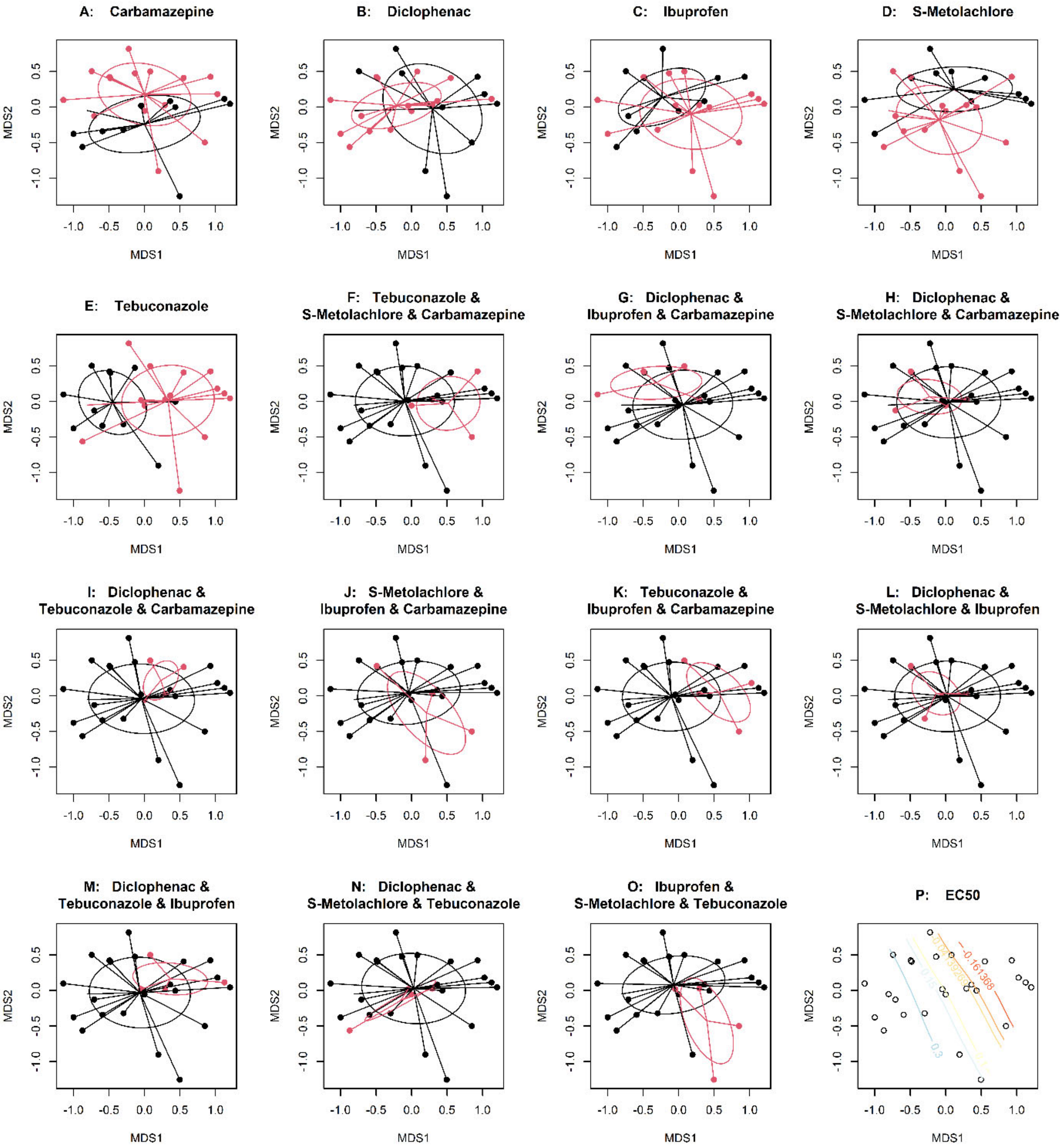

Figure S4. Non-Metric Multidimensional scaling ordinations based on Euclidean distances of transformed combination indices by compounds. (A) Carbamazepine; (B) Diclofenac; (C) Ibuprofen; (D) S-metolachlor; (E) Tebuconazole; (F) Ibuprofen & Diclofenac; (G) Tebuconazole & S-metolachlor & Carbamazepine; (H) Diclofenac & Ibuprofen & Carbamazepine; (I) Diclofenac & S-metolachlor & Carbamazepine; (J) Diclofenac & Tebuconazole & Carbamazepine; (K) S-metolachlor & Ibuprofen & Carbamazepine; (L) Tebuconazole & Ibuprofen & Carbamazepine; (M) Diclofenac & S-metolachlor & Ibuprofen; (N) Diclofenac & Tebuconazole & Ibuprofen; (O) Ibuprofen & S-metolachlor & Tebuconazole; (P) Contours of transformed combination index values at 50% effect level. Strength of synergy (blue) and antagonism (red) is shown by color and intensity.

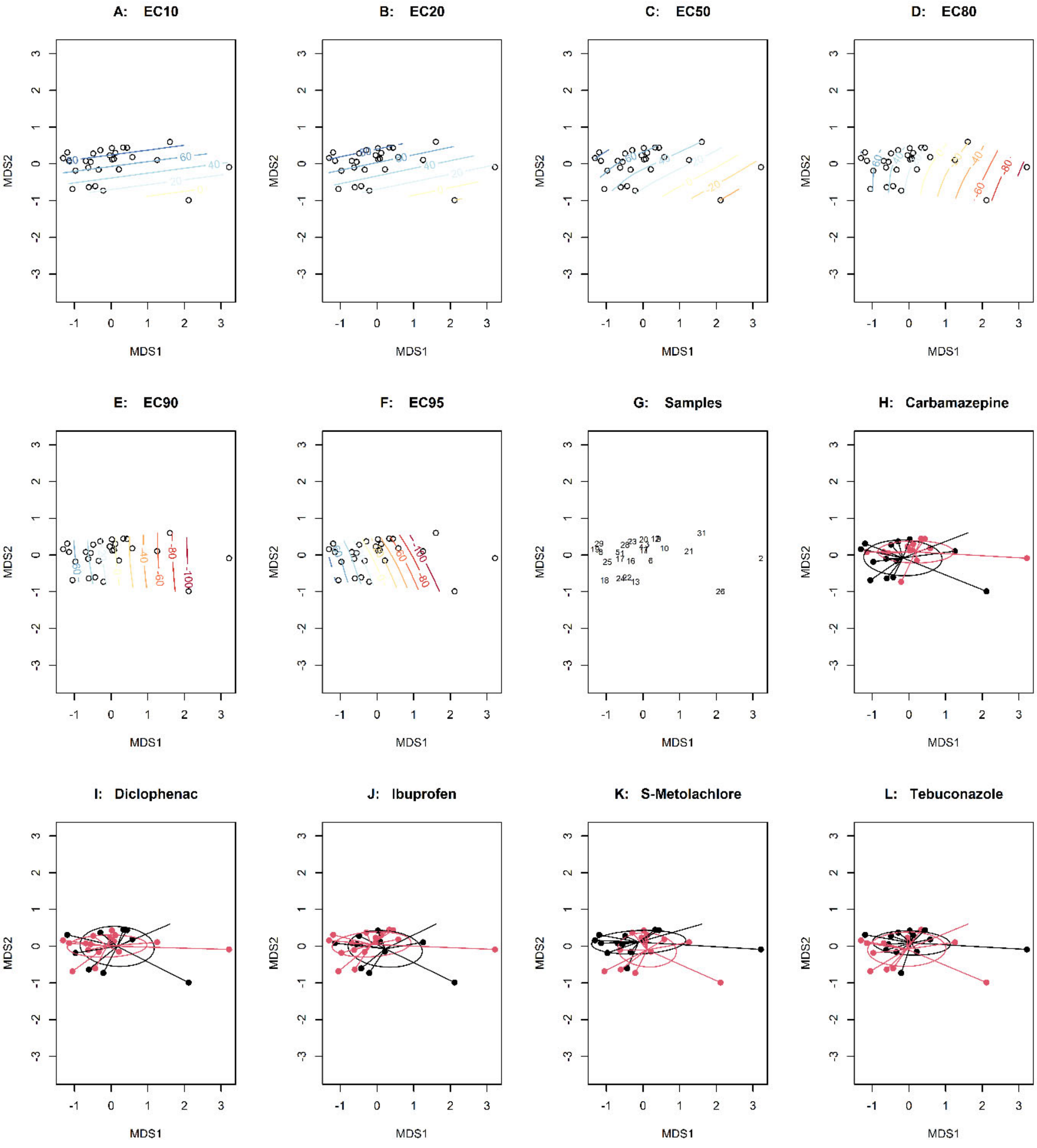

Figure S5. Effect level contours and compounds in Non-Metric Multidimensional scaling ordinations based on Euclidean distances of enhancement by Terbuthylazine. **(A)** Contours of enhancement by Terbuthylazine at 10% effect level. Strength of enhancement is shown by color and intensity, where blue is increase and red is decrease; **(B)** Contours of enhancement by Terbuthylazine at 20% effect level. Strength of enhancement is shown by color and intensity, where blue is increase and red is decrease; **(C)** Contours of enhancement by Terbuthylazine at 50% effect level. Strength of enhancement is shown by color and intensity, where blue is increase and red is decrease; **(D)** Contours of enhancement by Terbuthylazine at 80% effect level. Strength of enhancement is shown by color and intensity, where blue is increase and red is decrease; **(E)** Contours of enhancement by Terbuthylazine at 90% effect level. Strength of enhancement is shown by color and intensity, where blue is increase and red is decrease; **(F)** Contours of enhancement by Terbuthylazine at 95% effect level. Strength of enhancement is shown by color and intensity, where blue is increase and red is decrease; **(G)** Samples *. 1: Carbamazepine + Diclofenac + Terbuthylazine. 2: Carbamazepine + Diclofenac + Ibuprofen + Terbuthylazine. 3: Carbamazepine + Diclofenac + Ibuprofen + S-metolachlor + Terbuthylazine. 4: Carbamazepine + Diclofenac + Ibuprofen + S-metolachlor + Tebuconazole + Terbuthylazine. 5: Carbamazepine + Diclofenac + Ibuprofen + Tebuconazole + Terbuthylazine. 6: Carbamazepine + Diclofenac + S-metolachlor + Terbuthylazine. 7: Carbamazepine + Diclofenac + S-

metolachlor + Tebuconazole + Terbuthylazine. 8: Carbamazepine + Diclofenac + Tebuconazole + Terbuthylazine. 9: Carbamazepine + Ibuprofen + Terbuthylazine. 10: Carbamazepine + Ibuprofen + S-metolachlor + Terbuthylazine. 11: Carbamazepine + Ibuprofen + S-metolachlor + Tebuconazole + Terbuthylazine. 12: Carbamazepine + Ibuprofen + Tebuconazole + Terbuthylazine. 13: Carbamazepine + S-metolachlor + Terbuthylazine. 16: Diclofenac + Ibuprofen + Terbuthylazine. 17: Diclofenac + Ibuprofen + S-metolachlor + Terbuthylazine. 18: Diclofenac + Ibuprofen + S-metolachlor + Tebuconazole + Terbuthylazine. 19: Diclofenac + Ibuprofen + Tebuconazole + Terbuthylazine. 20: Diclofenac + S-metolachlor + Terbuthylazine. 21: Diclofenac + S-metolachlor + Tebuconazole + Terbuthylazine. 22: Diclofenac + Tebuconazole + Terbuthylazine. 23: Ibuprofen + S-metolachlor + Terbuthylazine. 24: Ibuprofen + S-metolachlor + Tebuconazole + Terbuthylazine. 25: Ibuprofen + Tebuconazole + Terbuthylazine. 26: S-metolachlor + Tebuconazole + Terbuthylazine. 28: Diclofenac + Terbuthylazine. 29: Ibuprofen + Terbuthylazine. 31: Tebuconazole + Terbuthylazine. * Excluded samples: 14: Carbamazepine + S-metolachlor + Tebuconazole + Terbuthylazine. 15: Carbamazepine + Tebuconazole + Terbuthylazine. 27: Carbamazepine + Terbuthylazine. 30: S-metolachlor + Terbuthylazine; **(H)** Carbamazepine; **(I)** Diclofenac; **(J)** Ibuprofen; **(K)** S-metolachlor; **(L)** Tebuconazole.

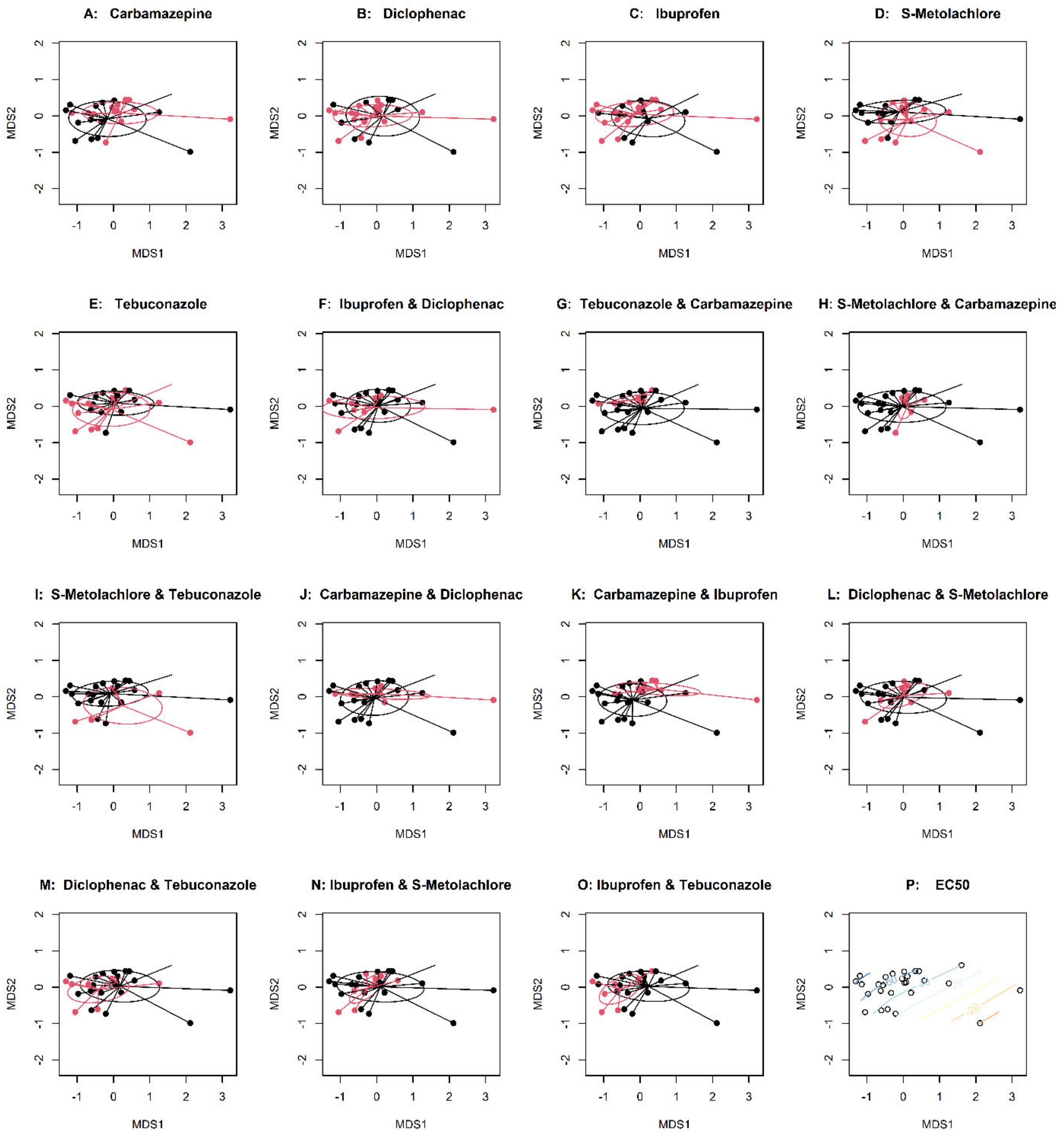

Figure S6. Non-Metric Multidimensional scaling ordinations based on Euclidean distances of enhancement by Terbuthylazine by compounds and compound combinations. (A) Carbamazepine; (B)

Diclofenac; (C) Ibuprofen; (D) S-metolachlor; (E) Tebuconazole; (F) Ibuprofen & Diclofenac; (G) Tebuconazole & Carbamazepine; (H) S-metolachlor & Carbamazepine; (I) S-metolachlor & Tebuconazole; (J) Carbamazepine & Diclofenac; (K) Carbamazepine & Ibuprofen; (L) Diclofenac & S-metolachlor; (M) Diclofenac & Tebuconazole; (N) Ibuprofen & S-metolachlor; (O) Ibuprofen & Tebuconazole; (P) Contours of enhancement by Terbuthylazine at 50% effect level. Strength of enhancement is shown by color and intensity, where blue is increase and red is decrease.

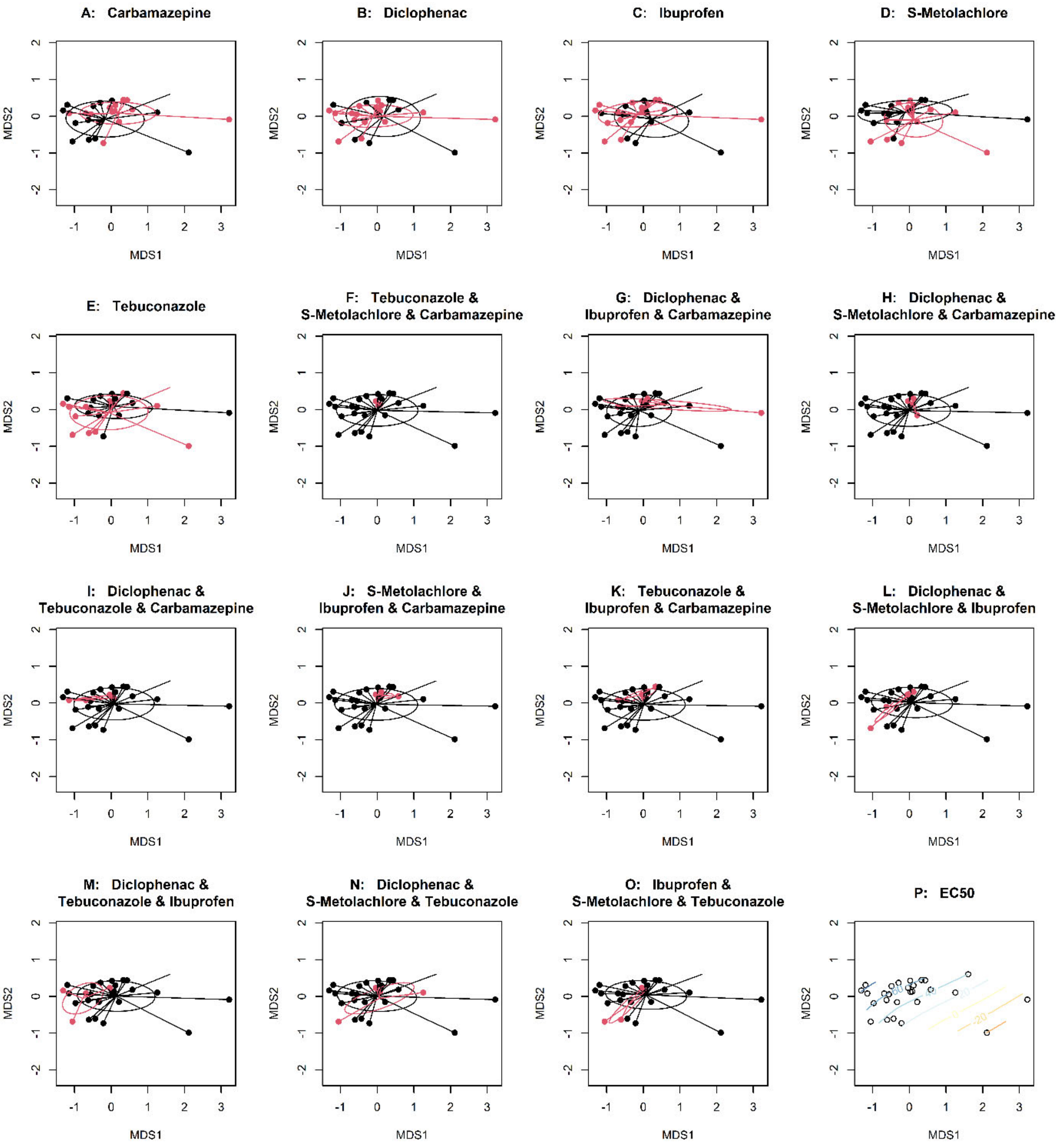

Figure S7. Non-Metric Multidimensional scaling ordinations based on Euclidean distances of enhancement by Terbuthylazine by compounds and compound combinations. A: Carbamazepine; B: Diclofenac; C: Ibuprofen; D: S-metolachlor; E: Tebuconazole; F: Tebuconazole & S-metolachlor & Carbamazepine; G: Diclofenac & Ibuprofen & Carbamazepine; H: Diclofenac & S-metolachlor & Carbamazepine; I: Diclofenac & Tebuconazole & Carbamazepine; J: S-metolachlor & Ibuprofen & Carbamazepine; K: Tebuconazole & Ibuprofen & Carbamazepine; L: Diclofenac & S-metolachlor & Ibuprofen; M: Diclofenac & Tebuconazole & Ibuprofen; N: Diclofenac & S-metolachlor & Tebuconazole; O: Ibuprofen & S-metolachlor & Tebuconazole; P: Contours of enhancement by Terbuthylazine at 50% effect level. Strength of enhancement is shown by color and intensity, where blue is increase and red is decrease.

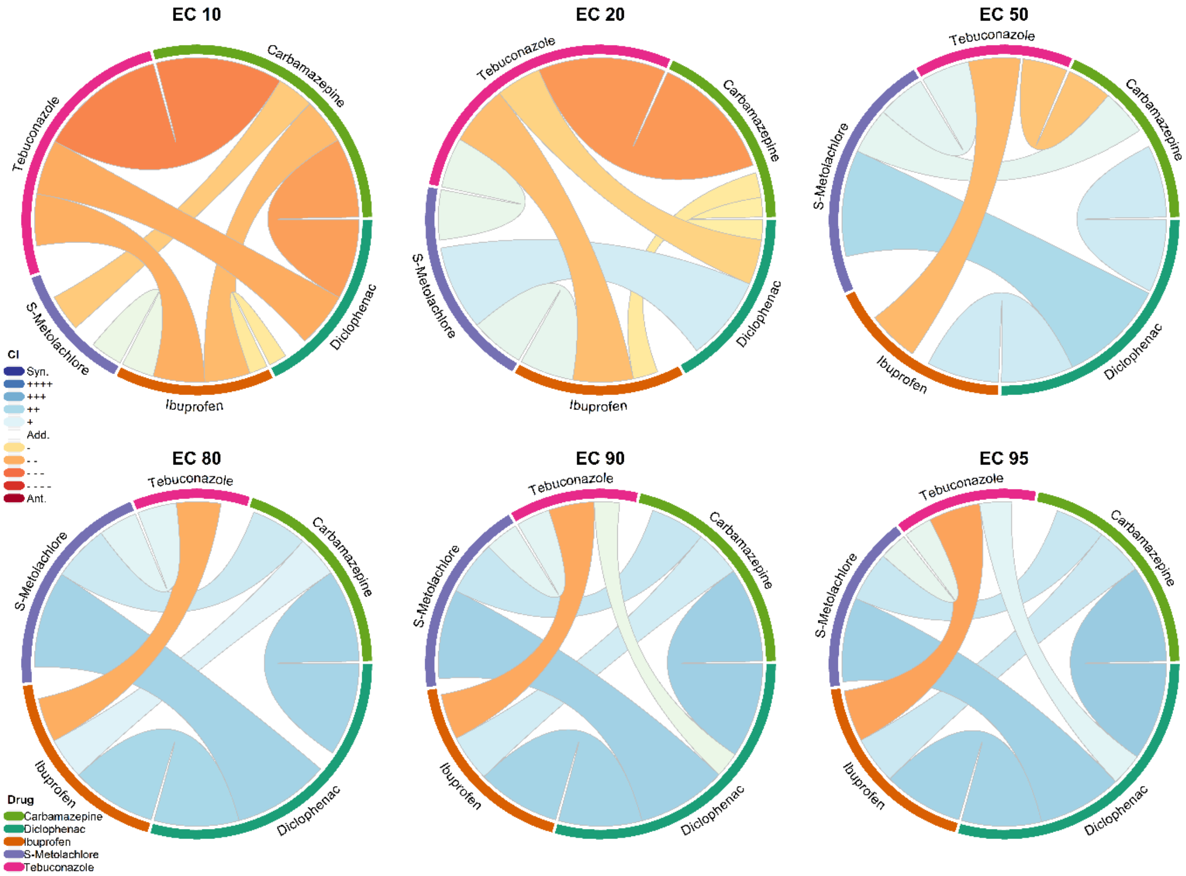

Figure S8. Weighted mean of transformed Combination Indexes of compound pairs from all the combinations where the chemicals were present in the examined combinations at effect sizes 10%, 20%, 50%, 80%, 90%, and 95%. The strength of synergy (blue) and antagonism (red) is shown by the width of linkage and color intensity.
